# Hippuric acid contributes to NK-cell activation with dependence on TLR2 in chronic hepatitis B complicated by MASLD

**DOI:** 10.1016/j.isci.2026.117362

**Published:** 2026-09-01

**Authors:** Luyuan Zhang, Anqi Li, Ke Xu, Siyang Tao, Meijuan Zheng

**Affiliations:** 1 Department of Clinical Laboratory, The First Affiliated Hospital of Anhui Medical University, Hefei, Anhui 230032, China

**Keywords:** chronic hepatitis B, MASLD, NK cells, toll-like receptor 2, hippuric acid

## Abstract

The coexistence of chronic hepatitis B and metabolic dysfunction-associated steatotic liver disease (CHB-MASLD) has been linked to accelerated fibrosis progression and less favorable clinical outcomes, but the immunopathological mechanisms remain incompletely understood. In the present study, we observed that CHB-MASLD patients exhibited significantly increased peripheral natural killer (NK) cell frequency and activation, with transcriptomic analysis revealing enrichment of inflammatory effector gene signatures. Notably, TLR2 was found to be upregulated in NK cells from CHB-MASLD patients and the frequency of TLR2-expressing NK cells correlated with metabolic parameters and liver stiffness. *In vitro*, metabolic-stress modeling increased TLR2 expression and enhanced NK-cell cytokine production. Untargeted plasma metabolomics identified hippuric acid as a candidate metabolite enriched in CHB-MASLD, and its abundance was associated with metabolic parameters and liver stiffness. Hippuric acid was involved in promoting NK-cell activation, and this effect was partially attenuated by TLR2 blockade. Furthermore, co-culture with hippuric acid-stimulated NK cells was associated with increased activation-related readouts in LX-2 cells, which were partially attenuated by TLR2 blockade. Collectively, these findings support an immunometabolic link among metabolic dysfunction, peripheral NK-cell activation, and a partially TLR2-dependent response to hippuric acid in CHB-MASLD, offering new insights into the immunopathology of CHB-MASLD.

## Highlights

• CHB-MASLD is associated with aggravated fibrosis-related clinical features
• Peripheral NK cells show enhanced activation features in CHB-MASLD
• TLR2 identifies a metabolically associated activated NK-cell subset
• Hippuric acid is linked to partially TLR2-dependent NK-cell activation

## Graphical abstract

**Figure:**
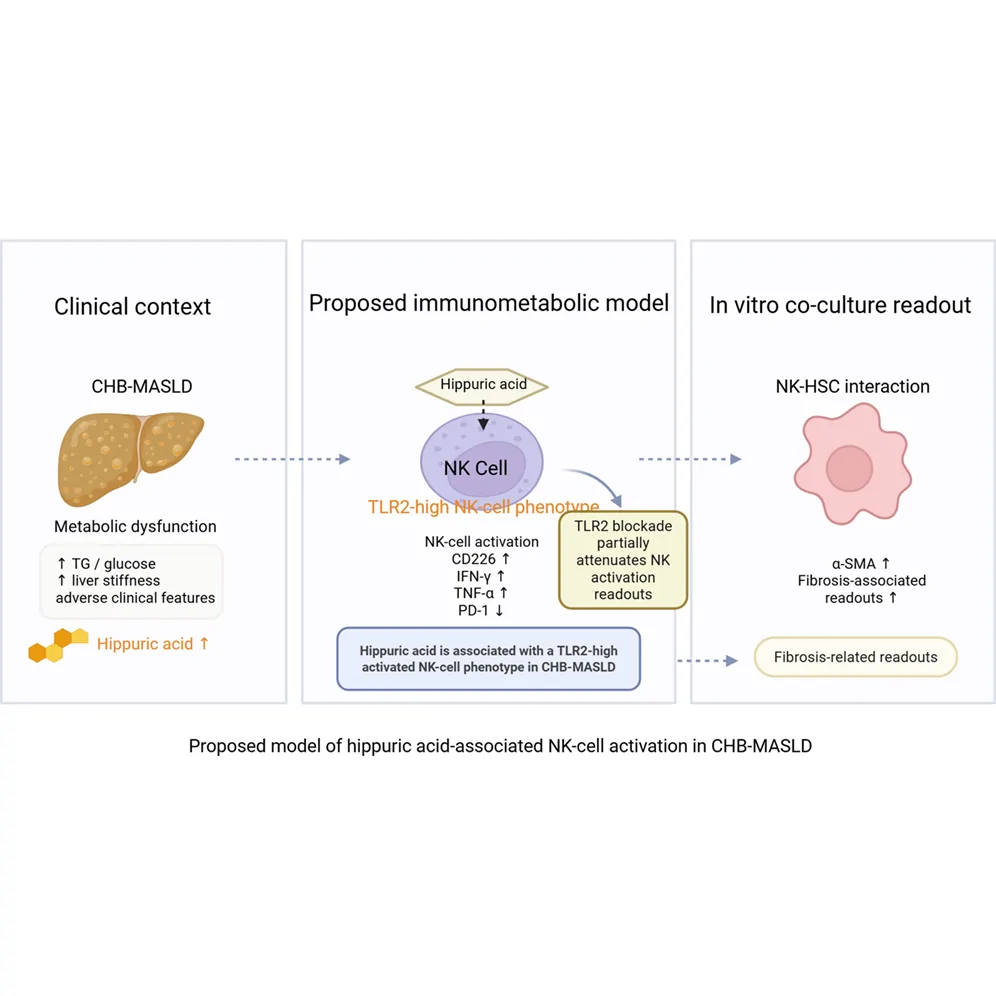

## Teaser

Biological sciences; Immunology; Cell biology

## Introduction

Chronic hepatitis B (CHB), caused by persistent hepatitis B virus (HBV) infection, remains a major cause of chronic liver disease and hepatocellular carcinoma worldwide. Progression of CHB is largely driven by sustained host immune-mediated liver injury, instead of direct viral cytopathicity alone.^1^^,^^2^ Metabolic dysfunction-associated steatotic liver disease (MASLD), formerly known as non-alcoholic fatty liver disease (NAFLD), is a leading cause of chronic liver disease worldwide. With increased prevalence of obesity and metabolic syndrome, the global prevalence of MASLD has risen sharply.^3^ Accumulating clinical evidence indicates that MASLD frequently coexists with CHB and is associated with accelerated fibrosis progression and poorer clinical outcomes.^4^^,^^5^ However, how the co-existence of metabolic dysfunction and CHB shapes hepatic immune responses remains incompletely understood.

Natural killer (NK) cells are central effectors of innate immunity and represent the first line of defense against many pathogens. Additionally, NK cells regulate liver inflammation and fibrosis.^6^^,^^7^ Liver-resident NK cells are recognized as frontline sensors of both immune and metabolic cues within the liver microenvironment.^7^^,^^8^ NK cell activation and effector functions are tightly linked to intracellular metabolic programs, and systemic metabolic perturbations can substantially reshape NK cell phenotypes and functional outputs.^6^^,^^9^^,^^10^ However, the specific mechanism in the context of CHB is still largely unexplored.

Innate immune pattern-recognition receptors (PRRs) have emerged as critical molecules linking metabolic stress to immune activation.^11^^,^^12^ PRR toll-like receptor 2 (TLR2) is expressed by NK cells and has been shown to regulate NK cell activation and cytokine production under inflammatory conditions.^11^^,^^13^^,^^14^ In metabolic liver disease, treatment with lipid-associated stressors such as palmitic acid (PA) can activate TLR2-dependent signaling pathways, promoting sterile inflammation relevant to steatohepatitis.^15^ Regarding CHB with metabolic dysfunction, existing studies have primarily focused on myeloid cells and hepatocytes but failed to clarify the contribution of the NK cell-intrinsic TLR2 signaling to immune remodeling.

Recent advances in metabolomics have unveiled that circulating metabolites are not merely biomarkers of metabolic status but function as bioactive signaling molecules capable of modulating innate immune responses. Both host- and microbiota-derived metabolites have increasingly been implicated in immune activation and inflammatory disease progression.^16^^,^^17^ Hippuric acid is a host-gut microbiota co-metabolite that has been consistently associated with metabolic health and systemic immune regulation.^17^^,^^18^^,^^19^ However, whether hippuric acid directly regulates NK cell activation or participates in immune remodeling in viral chronic liver disease remains to be investigated. It is hypothesized that the biological significance of hippuric acid may vary across disease contexts and requires further evaluation specifically in HBV-associated MASLD (CHB-MASLD).

This study sought to determine the association between metabolic dysfunction and NK-cell activation in CHB-MASLD and explore the potential role of metabolite-associated innate immune signaling pathways. By integrating clinical immune phenotyping, exploratory transcriptomic and metabolomic profiling, and *in vitro* functional modeling, this study aimed to define molecular features capable of distinguishing CHB-MASLD from CHB and to evaluate whether circulating metabolites, particularly hippuric acid, activate NK cells in a partially TLR2-dependent manner.

## Results

To improve clarity, the cohort allocation and sample size for each analysis are summarized in Figure S2 and Table S2. Not all of the analyses, including clinical assessment, flow cytometry, transcriptomics, metabolomics, and *in vitro* functional modeling, were performed in the same participants. Pre-specified data for each analysis were used according to sample availability, assay requirements, and data completeness. Correlation analyses were restricted to samples with complete paired measurements for the variables of interest.

### CHB-MASLD is associated with more severe fibrosis and exploratory adverse clinical outcomes

**Figure 1:**
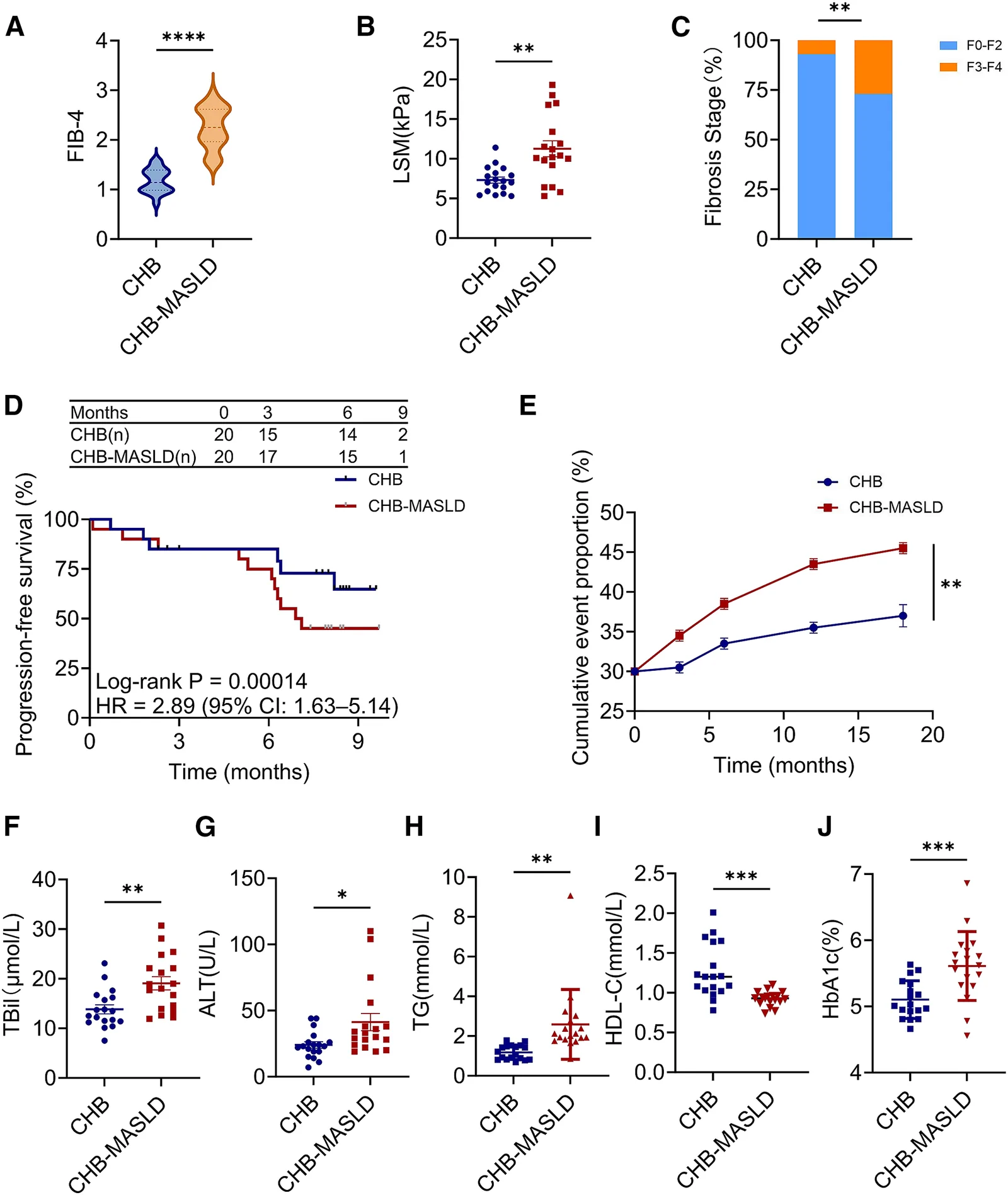
Metabolic dysfunction is associated with aggravated fibrosis-related indices and exploratory adverse clinical outcomes in CHB-MASLD (A) FIB-4 in patients with CHB (*n* = 18) and CHB-MASLD (*n* = 18). (B) Liver stiffness measurement (LSM, kPa) in patients with CHB (*n* = 18) and CHB-MASLD (*n* = 18). (C) Distribution of liver fibrosis stages (F0–F2 and F3–F4) in patients with CHB (*n* = 20) and CHB-MASLD (*n* = 20). (D) Kaplan-Meier analysis of progression-free survival in the exploratory follow-up cohort, including patients with CHB (*n* = 20) and CHB-MASLD (*n* = 20). Log rank *p* value and hazard ratio are indicated. (E) Cumulative incidence of liver-related events during follow-up in patients with CHB (*n* = 20) and CHB-MASLD (*n* = 20). (F–J) Comparisons of clinical and metabolic parameters between patients with CHB (*n* = 18) and CHB-MASLD (*n* = 18), including (F) TBil (μmol/L), (G) ALT (IU/L), (H) TGs (mmol/L), (I) HDL-C (mmol/L), and (J) HbA1c (%). Clinical comparisons were performed in the participants with complete baseline data. Fibrosis-related analyses were conducted in participants with available FIB-4 and LSM measurements. Outcome analyses were exploratory because of the limited sample size and follow-up duration. Data are presented as mean ± SD or median with interquartile range as appropriate. Statistical comparisons were performed using student’s *t* test or Mann-Whitney U test. Survival curves were analyzed using the log rank test. ∗*p* < 0.05, ∗∗*p* < 0.01, ∗∗∗*p* < 0.001, ∗∗∗∗*p* < 0.0001.

Patients with CHB-MASLD exhibited a significantly higher level of fibrosis-4 (FIB-4) and an increase in liver stiffness measurement (LSM) than patients with CHB, indicating more advanced liver fibrosis in CHB-MASLD (Figures 1A and 1B). Consistently, a higher proportion of CHB-MASLD patients showed advanced fibrosis, defined as fibrosis stage F3–F4 according to LSM-based staging criteria (Figure 1C).

In the exploratory follow-up cohort, patients with CHB-MASLD had shorter progression-free survival and a higher cumulative incidence of liver-related events than patients with CHB (Figures 1D and 1E).

Additionally, compared with CHB patients, CHB-MASLD patients presented with higher levels of total bilirubin (TBil) and alanine aminotransferase (ALT) (Figures 1F and 1G), together with pronounced metabolic abnormalities, including increased levels of triglyceride (TG) and glycated hemoglobin A1c (HbA1c) and a reduced level of high-density lipoprotein cholesterol (HDL-C) (Figures 1H–1J).

### Peripheral NK cells exhibit enhanced activation and effector programs in CHB-MASLD

**Figure 2:**
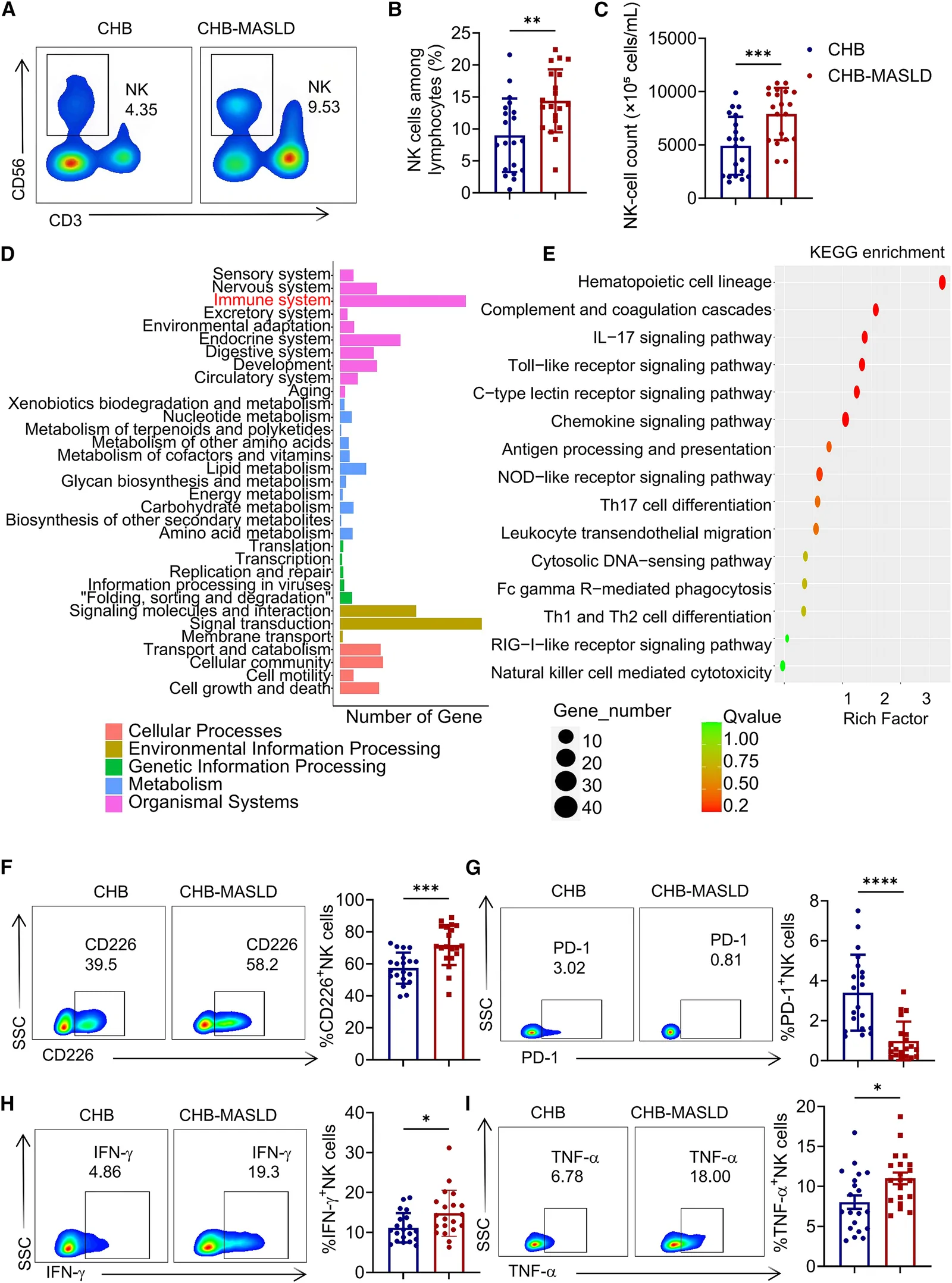
Enhanced activation and effector features of peripheral NK cells in patients with CHB-MASLD (A) Representative flow cytometry plots: gating strategy for identifying NK cells (CD3^−^CD56^+^) in peripheral blood from patients with CHB (*n* = 20) and CHB-MASLD (*n* = 20). (B) Frequency of NK cells among peripheral blood lymphocytes in patients with CHB (*n* = 20) and CHB-MASLD (*n* = 20). (C) Absolute NK-cell numbers (cellularity, cells/mL) in peripheral blood of patients with CHB (*n* = 20) and CHB-MASLD (*n* = 20). (D) KEGG classification of differentially expressed genes (DEGs) identified from exploratory transcriptomic profiling of sorted NK cells from patients with CHB (*n* = 4) and CHB-MASLD (*n* = 3). (E) KEGG pathway enrichment analysis of DEGs identified from the same exploratory RNA-seq cohort described in D. (F) Representative flow cytometry plots and quantification of CD226 expression in NK cells. (G) Representative flow cytometry plots and quantification of PD-1 expression in NK cells. (H and I) Intracellular cytokine production by NK cells, including (H) interferon-γ (IFN-γ) and (I) tumor necrosis factor-α (TNF-α). For (F–I), CHB (*n* = 20) and CHB-MASLD (*n* = 20). Flow cytometry was performed in an independent validation cohort with available PBMC samples and complete staining panels. Data are presented as mean ± SD. Statistical comparisons were performed using student’s *t* test or Mann-Whitney U test as appropriate.∗*p* < 0.05, ∗∗*p* < 0.01, ∗∗∗*p* < 0.001, ∗∗∗∗*p* < 0.0001.

To determine whether the metabolic dysfunction in CHB-MASLD modulates innate immune responses, peripheral NK cell abundance, phenotype, and function were assessed. In the flow cytometry validation cohort comprising patients with CHB (*n* = 20) and CHB-MASLD (*n* = 20), flow-cytometric analysis revealed a significantly higher proportion and absolute number of circulating NK cells in patients with CHB-MASLD than in patients with CHB (Figures 2A–2C).

To define the molecular programs underlying this phenotypic shift, transcriptomic profiling of NK cells was performed. Differentially expressed genes (DEGs) between groups were obtained and annotated by Kyoto Encyclopedia of Genes and Genomes (KEGG) pathways. It was found that the DEGs were predominantly enriched for immune-related functional categories (Figure 2D), including inflammatory signaling, immune activation, and innate immune pathways (Figure 2E).

Collectively, the metabolic dysfunction in CHB-MASLD was associated with a coordinated quantitative expansion and functional activation of peripheral NK cells and accompanied by enrichment of inflammatory effector programs.

### TLR2-expressing NK cells exhibit an activated phenotype associated with metabolic dysfunction in CHB-MASLD

**Figure 3:**
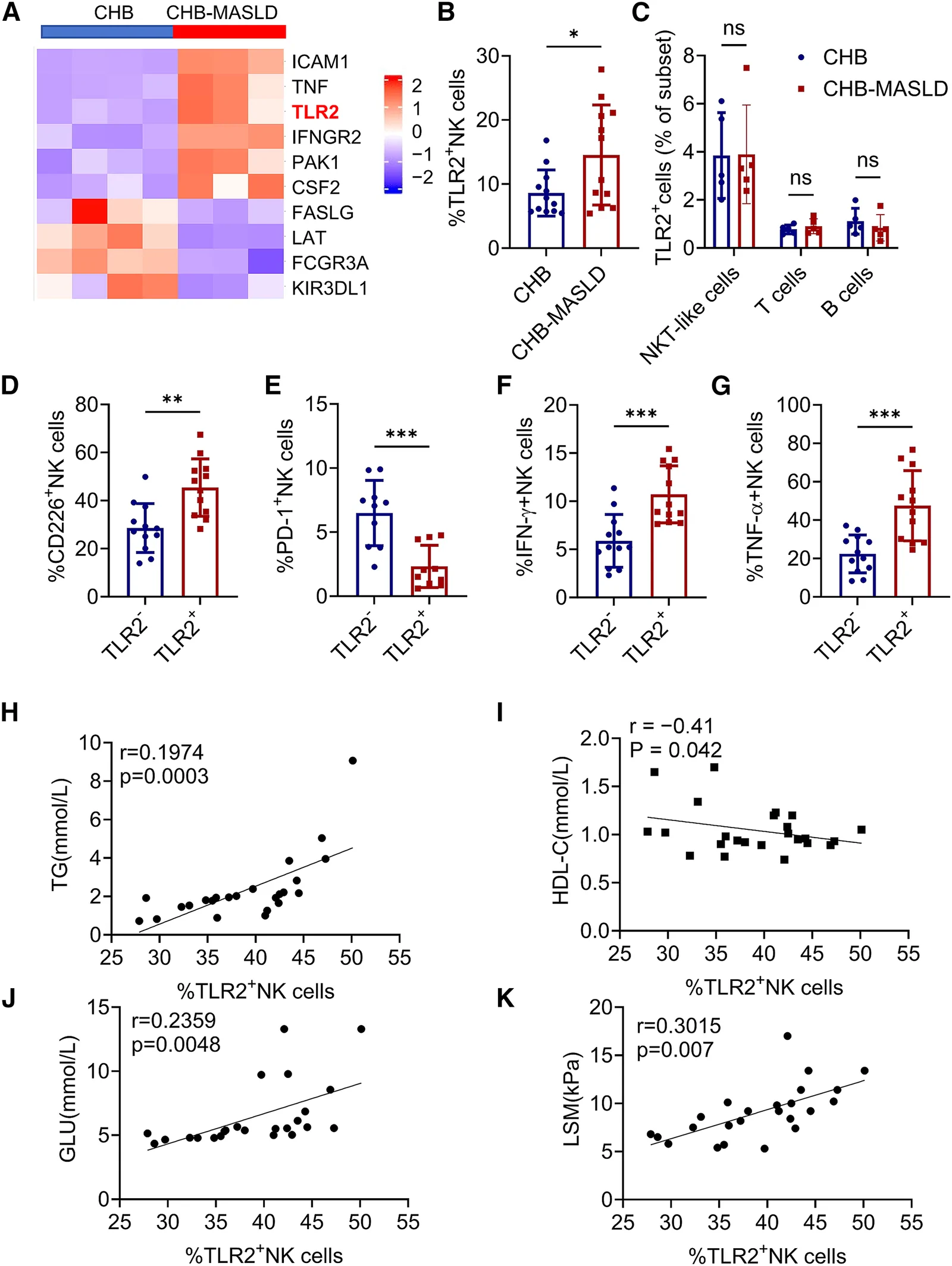
TLR2-expressing NK cells exhibit an activated phenotype associated with metabolic parameters and liver stiffness in CHB-MASLD (A) Heatmap: DEGs identified from exploratory transcriptomic profiling of sorted NK cells from patients with CHB (*n* = 4) and CHB-MASLD (*n* = 3), including TLR2. (B) Frequency of TLR2^+^ NK cells in peripheral blood from patients with CHB (*n* = 12) and CHB-MASLD (*n* = 12). (C) TLR2 expression in NK/T-like cells, T cells, and B cells from patients with CHB (*n* = 5) and CHB-MASLD (*n* = 5) with complete staining data. (D and E) Phenotypic comparison between TLR2^−^ and TLR2^+^ NK-cell subsets: (D) CD226 expression and (E) PD-1 expression. (F and G) Intracellular cytokine production in TLR2^−^ and TLR2^+^ NK cells: (F) IFN-γ and (G) TNF-α. (D–G) were generated from patients with CHB-MASLD (*n* = 12). (H–K) Correlation analyses between the frequency of TLR2^+^NK cells and metabolic or liver-related clinical parameters: (H) TG, (I) HDL-C, (J) GLU, and (K) LSM (*n* = 24 participants in total). TLR2-focused analyses were restricted to samples with complete TLR2 panel measurements, and correlation analyses were performed in the participants with complete paired clinical and flow cytometry data. Data are presented as mean ± SD. Statistical comparisons were performed using student’s *t* test or Mann-Whitney U test. Correlations were analyzed using Pearson correlation analysis. ∗*p* < 0.05, ∗∗*p* < 0.01, ∗∗∗*p* < 0.001.

To identify the molecular determinants of NK cell activation in CHB-MASLD, the transcriptomic profiles of NK cells from patients with CHB-MASLD and CHB were compared. Heatmap analysis revealed a distinct transcriptional pattern in the CHB-MASLD group, which was characterized by the upregulation of immune activation-related genes including TLR2 (Figure 3A).

Flow-cytometric validation showed a significantly higher frequency of TLR2^+^NK cells in peripheral blood of CHB-MASLD patients (Figure 3B), indicating the expansion of a TLR2-expressing NK-cell population in the presence of metabolic dysfunction. In contrast, TLR2 expression was not significantly altered in other lymphocyte subsets, including NK/T-like cells, T cells, and B cells (Figure 3C). Phenotypic profiling further demonstrated that TLR2^+^NK cells up-regulated the NK activating receptor CD226 and down-regulated the inhibitory receptor PD-1, consistent with a highly activated cellular state (Figures 3D and 3E).

In the combined cohort, the frequency of TLR2^+^ NK cells positively correlated with serum TG, blood glucose, and LSM, but negatively correlated with HDL-C level (Figures 3H–3K). These findings suggested that TLR2^+^ NK-cell frequency was associated with metabolic parameters and liver stiffness across the combined CHB and CHB-MASLD cohort.

Together, TLR2-expressing NK cells exhibited an activated phenotype in CHB-MASLD, and their frequency correlated with metabolic parameters and liver stiffness across the combined CHB and CHB-MASLD cohort. To further account for potential confounding factors, multivariable linear regression analyses were performed. CHB-MASLD status, TG, GLU, and LSM remained associated with TLR2^+^ NK-cell frequency after adjustment for prespecified covariates in exploratory multivariable analyses (Table S3).

### Metabolic-stress modeling increases TLR2 expression and NK-cell activation-related features

**Figure 4:**
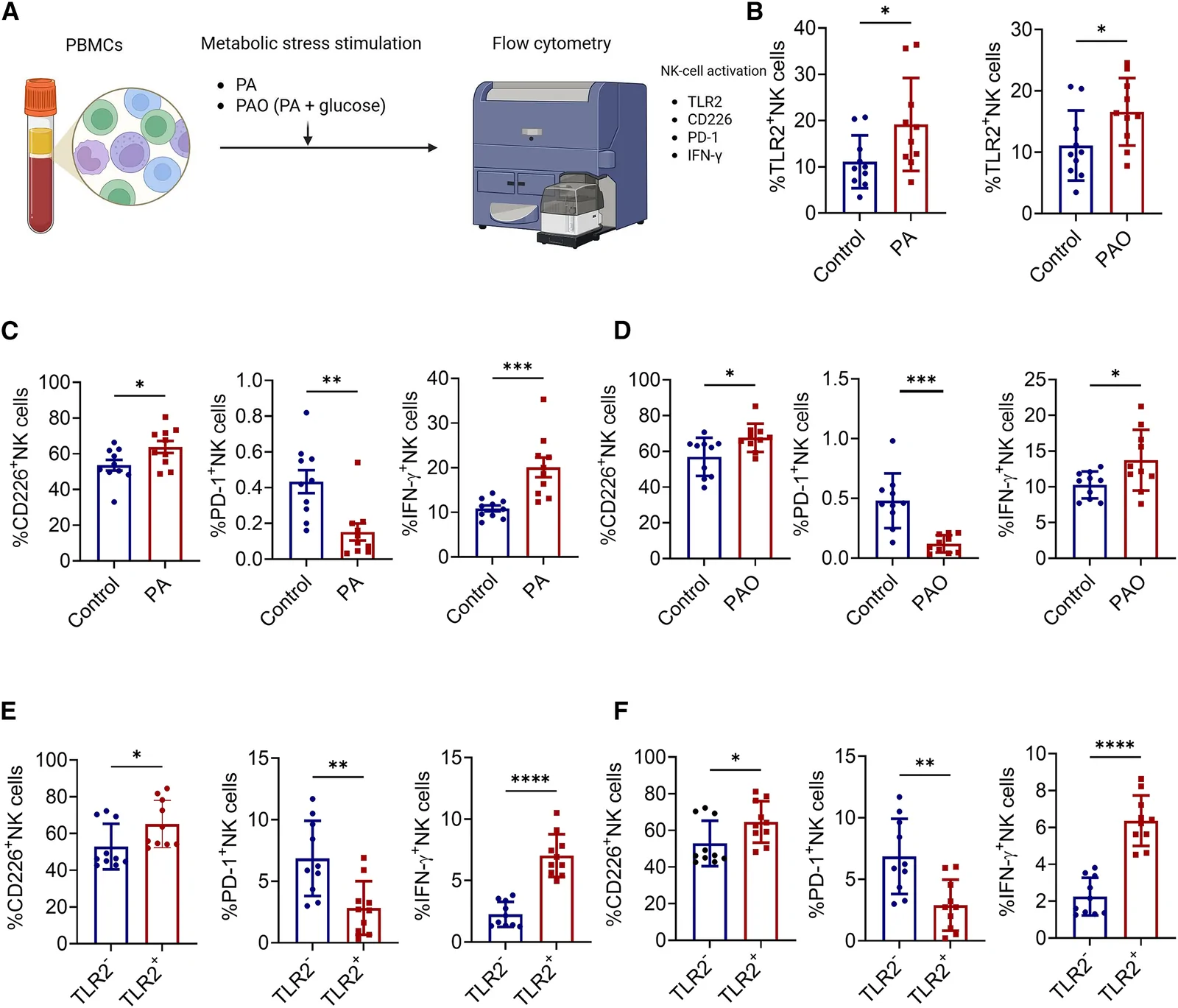
Metabolic-stress modeling increases TLR2 expression and NK-cell activation-related features *in vitro* (A) Schematic overview of the *in vitro* metabolic stress model. PBMCs were treated with PA and/or high glucose (HG) to model *in vitro* metabolic-stress conditions associated with CHB-MASLD. BSA-matched vehicle controls were used for PA stimulation, and mannitol was used as the osmotic control for high-glucose stimulation. Flow cytometry was performed to assess NK-cell activation and function (*n* = 10). (B) Flow cytometry analysis of TLR2 expression in NK cells following PA stimulation alone or in combination with HG (PAO). (C–F) Comparison of CD226, PD-1, and IFN-γ expression in NK cells: (C) following PA stimulation; (D) following PAO stimulation; (E) between TLR2^−^ and TLR2^+^ NK-cell subsets after PA treatment; and (F) between TLR2^−^ and TLR2^+^ NK-cell subsets after PAO treatment. *In vitro* metabolic-stress experiments were performed using PBMCs from an independent experimental subset of 10 donors (*n* = 10), as indicated in the figure labels. Each data point represents one independent donor sample. Technical replicate wells, where applicable, were averaged before statistical analysis. Data are presented as mean ± SD. Paired comparisons were performed using the paired student’s *t* test. ∗*p* < 0.05,∗∗*p* < 0.01,∗∗∗*p* < 0.001,∗∗∗∗*p* < 0.0001.

To examine whether metabolic stress is associated with NK-cell activation *in vitro*, peripheral blood mononuclear cells (PBMCs) were exposed to PA alone or in combination with D-glucose (PAO) for establishment of lipid and glucose overload (Figure 4A). Flow-cytometric analysis revealed that exposure to PA alone significantly increased the frequency of TLR2^+^NK cells, and the effect was further increased under the PAO condition (Figure 4B).

Consistently, PA-treated NK cells increasingly expressed the activating receptor CD226, produced more IFN-γ, and down-regulated PD-1 (Figure 4C). No significant alteration in NK-cell activation markers was observed in matched fatty acid-free bovine serum albumin (BSA) vehicle and mannitol osmotic controls, suggesting the specificity of metabolic stimulation conditions (Figure S4). In contrast, oleic acid stimulation produced only limited changes, with non-significant trends toward increased TLR2, CD226, and IFN-γ expression and reduced PD-1 expression (Figure S5). Notably, these NK-cell activation-associated changes were further pronounced under the PAO condition (Figure 4D), suggesting a greater effect of combined lipid and glucose stress on NK-cell activation programs.

Sub-group analysis was stratified by TLR2 expression (TLR2^+^/TLR2^−^). Under both the PA and PAO conditions, TLR2^+^NK cells displayed significantly higher CD226 expression, more IFN-γ production, and lower PD-1 expression compared with their TLR2^−^ counterparts (Figures 4E and 4F).

These findings collectively suggested that MASLD-related metabolic stress was associated with increased TLR2 expression and NK-cell activation-associated features *in vitro*, supporting a potential link between metabolic overload and altered NK-cell functional status.

### Plasma hippuric acid is elevated in CHB-MASLD and correlates with metabolic parameters and liver stiffness

**Figure 5:**
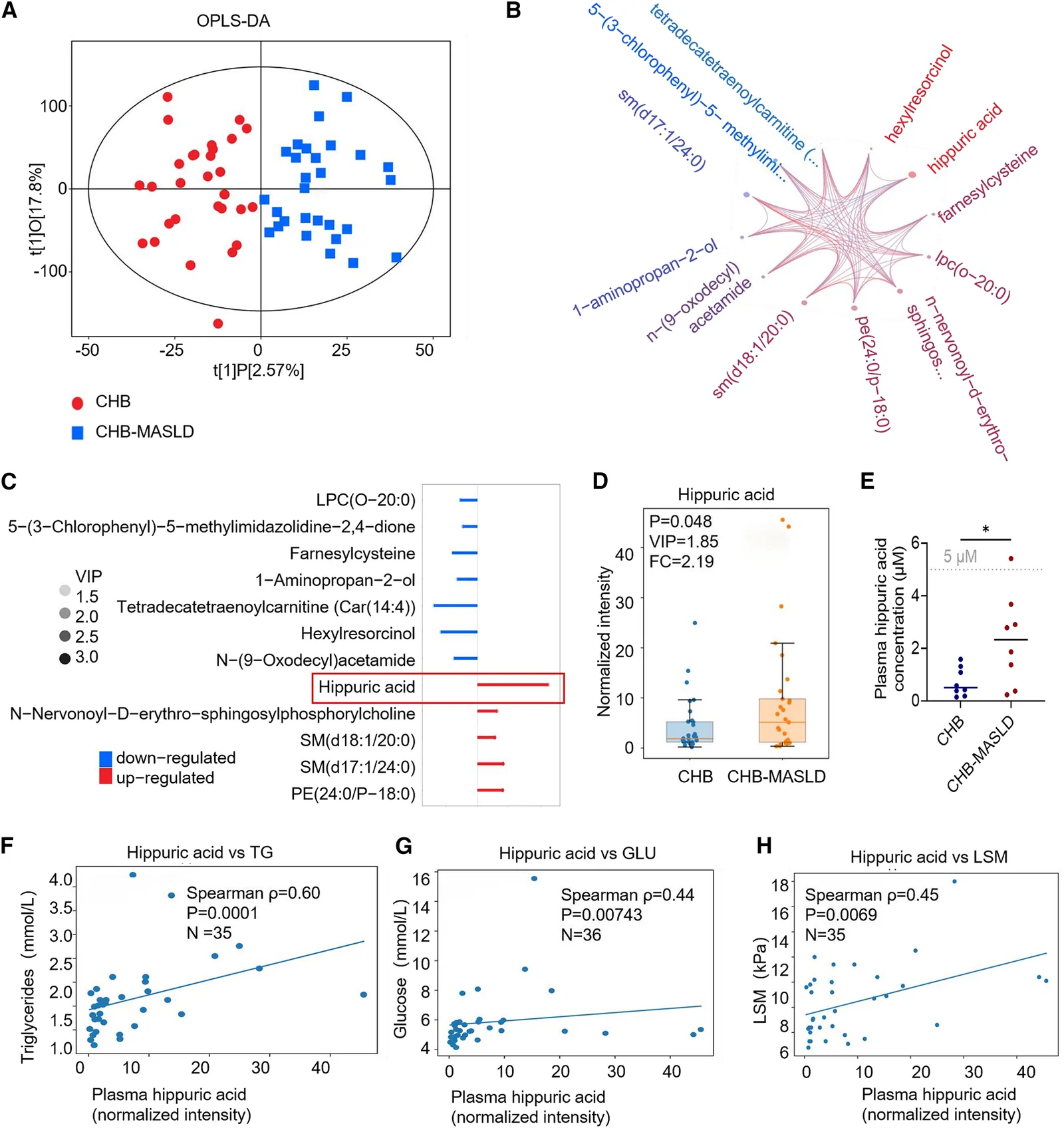
Plasma metabolomics identifies hippuric acid as a metabolite associated with CHB-MASLD (A) OPLS-DA score plot: separation of plasma metabolomic profiles between patients with CHB (*n* = 31) and CHB-MASLD (*n* = 29). (B) Network visualization of differentially abundant metabolites identified between the two groups. (C) Differential metabolite distribution based on variable importance in projection (VIP) scores and log_2_ fold change. Red indicates metabolites enriched in CHB-MASLD and blue indicates metabolites decreased in CHB-MASLD relative to CHB. (D) Comparison of normalized plasma hippuric acid levels between patients with CHB and CHB-MASLD. (A–D) were generated from the exploratory untargeted metabolomics cohort comprising patients with CHB (*n* = 31) and CHB-MASLD (*n* = 29). (E) Quantitative analysis of plasma hippuric acid concentrations in available plasma samples from patients with CHB (*n* = 8) and CHB-MASLD (*n* = 8). The dashed horizontal line indicates 5 μM. (F–H) Spearman correlation analyses between normalized plasma hippuric acid intensity and clinical parameters: (F) TG (*n* = 35), (G) GLU (*n* = 36), and (H) LSM (*n* = 35). Metabolomic profiling was performed in an exploratory plasma cohort, and correlation analyses were restricted to samples with complete paired metabolomic and clinical measurements. Data are presented as mean ± SD or median with interquartile range as appropriate. Statistical comparisons were performed using student’s *t* test or Mann-Whitney U test. Correlations were analyzed using Spearman correlation analysis. ∗*p* < 0.05.

To define MASLD-related metabolic features in CHB, untargeted metabolomic profiling of plasma was conducted. Orthogonal partial least squares discriminant analysis (OPLS-DA) revealed a clear separation between the CHB and CHB-MASLD groups, indicating a different global metabolic profile in the presence of metabolic dysfunction (Figure 5A). Differential metabolite analysis followed by validation with correlation network and pathway enrichment analyses identified a set of significantly altered metabolites contributing to group separation (Figures 5B and 5C).

Normalized plasma metabolite intensities were compared between groups. Notably, hippuric acid levels were significantly higher in patients with CHB-MASLD than in those with CHB (Figure 5D). These data identified hippuric acid as a candidate metabolite associated with CHB-MASLD. Quantitative measurement in an available subset of plasma samples further showed that the plasma concentration of hippuric acid was higher in patients with CHB-MASLD than in those with CHB and was largely within the low micromolar range (Figure 5E). Furthermore, Spearman correlation analysis revealed that the plasma level of hippuric acid positively correlated with systemic metabolic parameters (GLU, TG) and LSM (Figures 5F–5H), suggesting hippuric acid accumulation under both conditions of metabolic dysregulation and hepatic disease. Together, these findings identified elevated plasma hippuric acid as a candidate metabolic feature of CHB-MASLD and motivated subsequent functional experiments.

### Hippuric acid contributes to partially TLR2-dependent NK-cell activation and increased activation-related readouts in LX-2 cells

The plasma concentration of hippuric acid was higher in the CHB-MASLD group and was largely within the low micromolar range (Figure 5E), supporting the physiological relevance of the 5 μM stimulation condition. The 10 μM condition was used to assess dose responsiveness.

**Figure 6:**
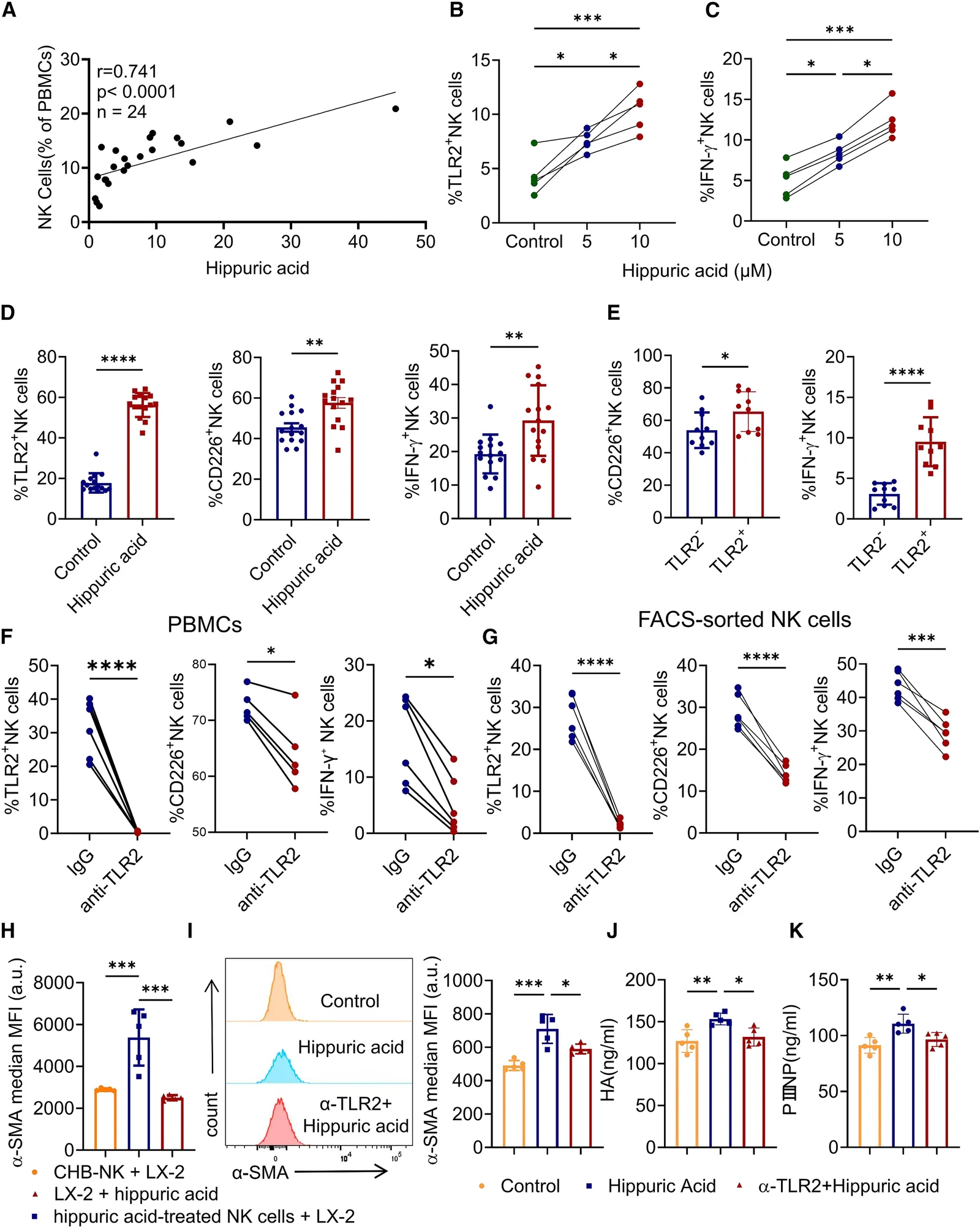
Hippuric acid is associated with partially TLR2-dependent NK-cell activation and increased LX-2 activation-related readouts (A) Pearson correlation between normalized plasma hippuric acid intensity and the proportion of NK cells among PBMCs in participants with complete paired measurements (*n* = 24). The line represents the simple linear regression fit. (B and C) Dose-dependent effects of hippuric acid stimulation at 0, 5, and 10 μM on NK-cell activation in PBMC cultures from 5 independent donors (*n* = 5): (B) frequency of TLR2^+^ NK cells and (C) percentage of IFN-γ-producing NK cells. Measurements were paired within donors. (D) Effect of hippuric acid stimulation on the frequencies of TLR2^+^ NK cells, CD226^+^ NK cells, and IFN-γ^+^ NK cells in PBMC cultures from 15 independent donors (*n* = 15). (E) Paired comparison of CD226 expression and IFN-γ production between TLR2^−^ and TLR2^+^ NK-cell subsets from 10 independent donors (*n* = 10). (F) PBMCs were pretreated with a TLR2-neutralizing antibody or an isotype control before hippuric acid stimulation. TLR2 blockade reduced the frequencies of TLR2^+^ NK cells, CD226^+^ NK cells, and IFN-γ^+^ NK cells in PBMC cultures from 6 independent donors (*n* = 6). (G) FACS-sorted NK cells were treated with a TLR2-neutralizing antibody or an isotype control, followed by hippuric acid stimulation. TLR2 blockade reduced TLR2 expression, CD226 expression, and IFN-γ production. Experiments were performed using cells from 5 independent donors (*n* = 5), with two technical replicate wells per condition. (H) Baseline and direct-exposure controls in the NK-LX-2 co-culture system. LX-2 cells were co-cultured with untreated CHB-derived NK cells or hippuric acid-treated NK cells, or were directly exposed to hippuric acid alone. α-SMA expression was quantified by flow cytometry. (I) Representative histogram and quantification of α-SMA expression in LX-2 cells after co-culture with control NK cells, hippuric acid-treated NK cells, or hippuric acid-treated NK cells subjected to TLR2 blockade. (J and K) Supernatant analysis of fibrogenic markers in the NK-LX-2 co-culture system: (J) hyaluronic acid (HA) and (K) procollagen III N-terminal peptide (PIIINP). Both markers were increased after hippuric acid stimulation and partially reduced following TLR2 blockade. (H–K) were generated using the same experimental cohort of NK cells from 5 independent donors (*n* = 5). PBMC stimulation assays, sorted NK-cell assays, and NK-LX-2 co-culture experiments were performed in independent experimental subsets. Unless otherwise specified, *n* represents the number of independent participants or donors. Technical replicate measurements were averaged to generate one value per biological sample and were not treated as independent observations. Data are presented as mean ± SD or median with interquartile range, as appropriate. Paired two-condition comparisons were performed using the paired student’s *t* test or Wilcoxon matched-pairs signed-rank test, as appropriate. Comparisons across three or more matched conditions were performed using one-way repeated-measures ANOVA with appropriate multiple-comparison testing or the Friedman test, as appropriate. ns, not significant; ∗*p* < 0.05; ∗∗*p* < 0.01; ∗∗∗*p* < 0.001; ∗∗∗∗*p* < 0.0001.

Normalized plasma hippuric acid intensity positively correlated with the proportion of NK cells in peripheral blood (Figure 6A). Hippuric acid stimulation dose-dependently increased the frequencies of TLR2^+^ NK cells and IFN-γ-producing NK cells (Figures 6B and 6C). It also increased the frequencies of TLR2^+^, CD226^+^, and IFN-γ^+^NK cells, while TLR2^+^ NK cells showed higher CD226 expression and IFN-γ production than TLR2^−^ NK cells (Figures 6D and 6E).

TLR2 blockade partially reduced hippuric acid-associated NK-cell activation in both PBMCs and FACS-sorted NK cells (Figures 6F and 6G). Pam3CSK4 stimulation further supported a functional association between TLR2-related signaling and NK-cell activation (Figure S6).

Hippuric acid-stimulated NK cells were associated with increased α-SMA expression in LX-2 cells, whereas direct hippuric acid exposure did not produce the same effect (Figure 6H). This increase was partially attenuated by TLR2 blockade (Figure 6I). HA and PIIINP levels were also increased and partially reduced following TLR2 blockade (Figures 6J and 6K). Similar findings were observed in the transwell system (Figure S3).

## Discussion

Despite advances in antiviral therapy, HBV infection remains a major cause of cirrhosis and hepatocellular carcinoma worldwide.^1^^,^^2^^,^^20^ Increasing epidemiological evidence has indicated that MASLD frequently coexists with CHB and is associated with fibrosis progression and adverse clinical outcomes.^4^^,^^5^ Consistently, the present study found that patients with CHB-MASLD presented with more severe fibrosis-related features and less favorable exploratory clinical outcomes than those with CHB alone. It was found that CHB-MASLD correlated with higher fibrosis indices, increased liver stiffness, and reduced event-free survival. These findings support the concept that MASLD is not merely a parallel metabolic comorbidity but may influence the clinical trajectory of CHB. More broadly, our results highlight the importance of metabolic context for understanding the immune regulation in CHB. In viral chronic liver disease, metabolic disturbance may reshape immune responses and contribute to heterogeneous disease progression.

From an immunological perspective, CHB-MASLD was characterized by quantitative expansion and functional activation of peripheral NK cells. Compared with CHB, CHB-MASLD correlated with increased NK-cell frequency and cellularity. Additionally, CHB-MASLD showed enrichment for inflammatory effector pathways. Functional analysis revealed that the NK cells from CHB-MASLD patients showed enhanced activation phenotypes, which were manifested by enhanced CD226 expression, elevated cytokine production (IFN-γ and TNF-α), and reduced PD-1 expression. It has been established that NK-cell function is metabolically regulated, and systemic metabolic stress can modify NK-cell phenotypes.^8^^,^^10^^,^^21^^,^^22^ NK-cell dysregulation has been reported in both chronic viral infection and metabolic liver disease alone but remains insufficiently defined under a background of HBV infection complicated by metabolic dysfunction. Our findings suggest that metabolic dysfunction was associated with the coordinated transcription and activation of NK cells, supporting emerging models in which immune cells integrate metabolic and inflammatory cues to determine effector states.

It should be emphasized that the immune profiling in this study was primarily performed in peripheral blood. Circulating NK cells may not fully recapitulate liver-resident NK-cell phenotypes, especially as the liver microenvironment contains unique cytokine, metabolic, stromal, and antigenic signals that may modify TLR2 expression and hippuric acid responsiveness. In this context, findings from the present study should be interpreted as evidence of peripheral NK-cell remodeling in CHB-MASLD, rather than as direct proof of intrahepatic NK-cell behavior. Future studies using liver tissue, spatial profiling, or paired blood-liver analyses will be required to determine whether similar TLR2^+^NK-cell programs operate within the hepatic compartment.

Through transcriptomic and phenotypic analyses, this study focused on NK-cell receptor TLR2 for further analysis. Differential expression analysis revealed a prominent upregulation of TLR2 expression in CHB-MASLD patients. Further analysis found that TLR2 expression defined a distinct NK-cell subset characterized by enhanced effector activity, which was manifested by increased CD226 expression and elevated cytokine production. Importantly, the frequency of TLR2^+^NK cells was found to correlate positively with metabolic parameters and liver stiffness and negatively with protective metabolic indices. TLR2-associated signaling has been widely implicated in metabolic inflammation, particularly in myeloid and parenchymal cells.^13^^,^^23^^,^^24^^,^^25^ There is evidence that NK cells can express TLR family receptors and respond to TLR-dependent cues. However, the cell-intrinsic role of TLR2 programs in NK cells in viral chronic liver disease complicated by metabolic dysfunction has not been well characterized.^11^^,^^13^^,^^14^ Our results extend previous observations by suggesting that TLR2 expression may delineate a metabolically responsive NK-cell subset that is associated with the systemic metabolic burden and disease severity in CHB.

To further explore how metabolic dysfunction influences NK-cell behavior, metabolic stress was established *in vitro* through lipid and glucose stimulation. Exposure to PA or PAO led to increased TLR2 expression and NK-cell activation features, recapitulating key immune features observed in CHB-MASLD patients. Additionally, CD226 was increasingly expressed in NK cells under stimulation, and more IFN-γ was produced. These findings are consistent with prior studies, where metabolic stressors were found to activate inflammatory signaling through innate immune receptors.^12^^,^^15^^,^^26^ These data also suggest that NK cells may be responsive to metabolic stress, which extends previous work on macrophages, dendritic cells, and hepatocytes.^25^^,^^27^ Moreover, these observations support the emerging concept that innate immune cells can function as metabolically responsive immune entities in addition to traditional immune sentinels.^24^^,^^28^

Metabolomic analyses provided further insight into the metabolic signals underlying these immune alterations. Among the circulating metabolites differentially altered in CHB-MASLD, hippuric acid was enriched in patients with metabolic dysfunction and associated with liver stiffness and metabolic parameters. Previous studies have linked hippuric acid or hippurate levels to metabolic health, microbiome composition, and systemic metabolic state.^17^^,^^29^ Microbiota-derived metabolites were historically regarded as a metabolic biomarker. However, accumulating evidence has indicated their active role in immune regulation and inflammatory disease progression.^30^^,^^31^ In this study, the circulating hippuric acid level was observed to correlate with NK-cell activation in CHB-MASLD patients, suggesting that metabolic intermediates may contribute to immune remodeling in viral chronic liver disease.

These findings should be interpreted alongside recent studies. It has been reported that hippuric acid may exert protective or compensatory effects in non-viral MASLD; additionally, hippuric acid can potentiate innate immune inflammation through TLR-MyD88 signaling in macrophages.^18^^,^^29^ Hippuric acid, therefore, is unlikely to represent a universal pro-inflammatory metabolite. In this study, hippuric acid was identified as a candidate metabolite associated with peripheral NK-cell activation in CHB-MASLD.

The role of hippuric acid should be interpreted in a disease-context-dependent manner. Previous studies in non-viral metabolic liver disease linked hippuric acid to metabolic health or compensatory host-microbiota responses. Conversely, the present study focused on CHB complicated by MASLD. The differences in viral background, antiviral treatment, gut-liver axis status, cohort composition, analytical platform, and disease stage can partly explain the discrepancy in results across studies. In this context, our results should not be interpreted as a demonstration that hippuric acid is a universal pro-inflammatory metabolite or a direct TLR2 ligand. The main contribution of this study is to suggest that hippuric acid may serve as a candidate metabolite associated with partially TLR2-dependent NK-cell activation in CHB-MASLD, through integration of multiple analytical methods (clinical phenotyping, NK-cell immune profiling, exploratory transcriptomics, untargeted metabolomics, and *in vitro* TLR2 blockade experiment). Moreover, quantitative measurement was performed and further showed that the circulating hippuric acid level in CHB-MASLD was largely within the low micromolar range, supporting the relevance of the lower *in vitro* stimulation condition. The higher 10 μM stimulation condition should be interpreted as a dose-response exposure rather than a direct replication of circulating concentration.

*In vitro* functional experiments demonstrated that hippuric acid stimulation was associated with increased NK-cell activation and cytokine production. However, this effect was partially attenuated by TLR2 blockade, supporting a partially TLR2-dependent component to hippuric acid-associated NK-cell activation. These findings align with emerging immunometabolic paradigms that circulating metabolites function as signaling molecules to modulate immune cell behavior.^16^^,^^27^ Our study also found that co-culture of LX-2 and hippuric acid-stimulated NK cells increased α-SMA expression and secretion of matrix-related markers HA and PIIINP, and these effects were partially attenuated by TLR2 blockade. This finding should be interpreted in the context of a simplified *in vitro* co-culture system. Additionally, it does not contradict the established anti-fibrotic roles of NK cells *in vivo*. Evidence shows that the effect of NK cells on HSCs is highly dependent on activation intensity, cytokine milieu, cell-contact signals, and disease stage.^7^ Collectively, these observations support an immunometabolic framework, whereby metabolic cues influence innate immune activation and LX-2 activation-related readouts in a partially TLR2-dependent manner. From a clinical perspective, these findings may provide a possible immunometabolic context for understanding why CHB complicated by MASLD is associated with less favorable outcomes and suggest that integrating metabolic parameters into immune risk-stratification models may improve clinical prediction.

In summary, metabolic dysfunction was associated with innate immune regulation during chronic HBV infection rather than acting as a parallel metabolic comorbidity. A metabolically responsive TLR2^+^NK-cell subset associated with systemic metabolic burden and disease severity was identified, and hippuric acid level was found to correlate with NK-cell activation in a partially TLR2-dependent manner. These observations highlight a potential immunometabolic pathway linking metabolic dysfunction with NK-cell activation and LX-2 activation-related readouts *in vitro*. Future studies integrating longitudinal clinical cohorts, mechanistic signaling analyses, and *in vivo* models will be important to determine whether metabolite-TLR2-associated responses can be therapeutically modulated in CHB.

### Limitations of the study

This study has several limitations. First, the current study had a moderate sample size, despite the integration of multiomics approaches and functional validation. Future larger, multicenter longitudinal cohorts are required to confirm our findings. Second, our mechanistic insights were primarily derived from *in vitro* studies on PBMCs and sorted NK cells, which do not fully capture the complexity of the liver immune microenvironment. Third, this study did not demonstrate the direct binding between hippuric acid and TLR2. In addition, receptors other than TLR2 or parallel signaling pathways may contribute to hippuric acid-associated NK-cell activation. Fourth, our metabolomic analysis focused on hippuric acid as a candidate immunometabolic mediator but did not systematically explore broader metabolite-immune interaction networks. Finally, the immune profiling was primarily performed in peripheral blood, necessitating further exploration of the relationship between circulating and liver-resident NK cell states in tissue-based and *in vivo* models. Although sex was included as a covariate in multivariable analyses, the study was not designed or powered to evaluate sex- or gender-specific effect modification; therefore, the influence of sex and gender on the observed associations remains uncertain.

## Resource availability

### Lead contact

Requests for further information and resources should be directed to and will be fulfilled by the lead contact, Meijuan Zheng (zhengmj@fy.ahmu.edu.cn).

### Materials availability

This study did not generate new unique reagents.

### Data and code availability

• The RNA-seq data generated in this study have been deposited in GEO under accession number GSE325645.
• The metabolomics dataset has been deposited in OMIX and is publicly available under accession number OMIX015709.
• This study does not report original code.
• Any additional information required to reanalyze the data reported in this study is available from the lead contact upon reasonable request.

## Acknowledgments

This work was supported by the 10.13039/501100001809National Natural Science Foundation of China (grant number 92269109) and the Outstanding Youth Project of the Scientific Research Foundation of the Education Department of Anhui Province of China (grant number 2023AH020048).

## Author contributions

Conceptualization, methodology, validation, formal analysis, investigation, resources, data curation, writing – original draft, writing – review and editing, visualization, and project administration, L.Z.; investigation, resources, and writing – review and editing, A.L.; validation, visualization, and writing – review and editing, K.X. and S.T.; conceptualization, methodology, resources, supervision, project administration, writing – review and editing, and funding acquisition, M.Z.

## Declaration of interests

The authors declare no competing interests.

## STAR★Methods

### Key resources table

**Table:** 

REAGENT or RESOURCE	SOURCE	IDENTIFIER
**Antibodies**		

APC-R700 anti-CD3	BioLegend	Cat# 317340; RRID: AB_2563408
APC-H7 anti-human CD45	BD Biosciences	Cat# 563094; RRID: AB_2738001
PerCP-Cy5.5 anti-CD45	BD Biosciences	Cat# 340953; RRID: AB_400194
BV510 anti-human CD56	BD Biosciences	Cat# 563041; RRID: AB_2732786
BV421 anti-human CD56	BD Biosciences	Cat# 562751; RRID: AB_2732054
PE-Cy7 anti-CD56	BD Biosciences	Cat# 557747; RRID: AB_396853
FITC anti-human TLR2	BioLegend	Cat# 309705; RRID: AB_314775
APC anti-human CD226	BioLegend	Cat# 338311; RRID: AB_2561951
FITC anti-human CD226	BD Biosciences	Cat# 559788; RRID: AB_397329
PE anti-human PD-1	BD Biosciences	Cat# 560795; RRID: AB_2033989
PerCP-Cy5.5 anti-IFN-γ	BioLegend	Cat# 502525; RRID: AB_961357
APC anti-human TNF-α	BioLegend	Cat# 502913; RRID: AB_315265
PE anti-human TNF-α	BD Biosciences	Cat# 559321; RRID: AB_397219
FITC anti-human TNF-α	BD Biosciences	Cat# 340511; RRID: AB_400434
PE anti-human IgG1 isotype control	BD Biosciences	Cat# 555749; RRID: AB_396091
α-SMA antibody	Abcam	Cat# ab7817; RRID: AB_262054
Anti-TLR2 neutralizing antibody (MAb-mTLR2, clone T2.5)	InvivoGen	Cat# mab2-mtlr2

**Chemicals, Peptides, and Recombinant Proteins**		

Pam3CSK4	InvivoGen	Cat# tlrl-pms
Human whole-blood mononuclear cell separation medium	TBD Science	Cat# LDS1075
Hippuric acid	Source Leaf	Cat# B25661
Palmitic acid (PA)	Kunchuang	Cat# KC004
Oleic acid	Kunchuang	Cat# KC005
D-glucose	Biosharp	Cat# BS099-500g
Mannitol	MCE	Cat# HY-N0378
Monensin	MultiSciences Biotech	Cat# CS0004
7-AAD	BioLegend	Cat# 420403
Recombinant human IL-12	PeproTech	Cat# 200-12
Recombinant human IL-15	PeproTech	Cat# 200-15

**Deposited Data**		

RNA-seq dataset, this paper	GEO	GSE325645
Metabolomics dataset, this paper	OMIX	OMIX015709

**Experimental Models: Cell Lines**		

Human hepatic stellate cell line (LX-2)	BeNa Culture Collection (BNCC)	Cat# BNCC337957

**Software and Algorithms**		

FlowJo v10.8.2	BD Biosciences	RRID: SCR_008520
GraphPad Prism 10	GraphPad Software	RRID: SCR_002798
R software	R Foundation	RRID: SCR_001905
HISAT2 v2.0.4	Johns Hopkins University	RRID: SCR_015530
DESeq2	Bioconductor	RRID: SCR_015687
XCMS-based in-house R pipeline	Biotree	N/A
BiotreeDB	Biotree	N/A

### Experimental model and study participant details

#### Human participants and clinical data collection

Participants were adult CHB patients consecutively enrolled at the First Affiliated Hospital of Anhui Medical University between April 2024 and February 2026. Participants were stratified into two groups, CHB and CHB-MASLD, according to the absence/coexistence of MASLD. CHB was defined by persistent positivity of HBsAg for more than 6 months^32^ MASLD was diagnosed by the presence of hepatic steatosis and at least one cardiometabolic risk factor, according to current international consensus criteria.^3^ Group allocation was observational and based on predefined diagnostic criteria; no random allocation was performed.

Inclusion criteria: (1) aged 18 years or older; (2) complete clinical data; (3) blood samples available for analysis. Exclusion criteria: (1) viral coinfection, autoimmune or alcoholic liver disease, drug-induced liver injury, malignancy, severe systemic inflammatory disease; (2) pregnancy; (3) prior or current immunosuppressive treatment; (4) incomplete key clinical records.

Baseline demographic, virological, metabolic, and liver-related clinical variables were collected from medical records at enrollment, including age, sex, BMI, ALT, aspartate aminotransferase (AST), TBil, albumin, platelet count, HBV DNA, HBeAg status, antiviral therapy, fasting glucose, HbA1c, TG, HDL-C, FIB-4,^33^ and LSM, where available. The follow-up schedule and censoring rules are summarized in Table S4. Baseline characteristics of the enrolled patients are summarized in Table S1. The study protocol was approved by the Ethics Committee of the First Affiliated Hospital of Anhui Medical University (Approval No. PJ2026-02-34). Written informed consent was obtained from all participants prior to enrollment. All procedures involving human participants were performed in accordance with the ethical principles of the Declaration of Helsinki. All participants were adults (≥18 years); age and sex distributions for the baseline clinical cohort are provided in Table S1.

#### Cohort allocation and sample-size definition

A total of 36 patients were included in the updated baseline clinical cohort (CHB, *n* = 18; CHB-MASLD, *n* = 18). Fibrosis-stage distribution analysis was performed in a separate subset comprising patients with CHB (*n* = 20) and CHB-MASLD (*n* = 20). Different subsets of the overall study population were selected as needed for specific analyses, including follow-up/outcome assessment, flow cytometry, TLR2-focused flow cytometry, RNA-seq, metabolomic profiling, and *in vitro* experiments.

Because complete clinical variables, PBMC samples, RNA preparations, plasma samples, and follow-up data were not available for all participants, different prespecified cohorts or experimental subsets were used for individual analyses according to sample availability, assay requirements, and data completeness. Baseline clinical comparisons, fibrosis-related analyses, follow-up outcome analyses, flow cytometry, TLR2-focused flow cytometry, exploratory RNA-seq, exploratory untargeted metabolomics, and *in vitro* functional experiments were therefore performed in separate cohorts or independent experimental subsets. The cohort allocation and sample size for each analysis are summarized in Figure S2 and Table S2.

#### LX-2 HSCs

The human HSC line LX-2 was obtained from BeNa Culture Collection (BNCC, Beijing, China; Cat. No. BNCC337957). The identity of the cell line was authenticated with short tandem repeat (STR) profiling according to the authentication report provided by the supplier. Cells were routinely tested for mycoplasma contamination and confirmed to be mycoplasma-negative before experiments. LX-2 cells were maintained in high-glucose Dulbecco’s modified Eagle’s medium (DMEM; Gibco, USA) supplemented with 10% fetal bovine serum (FBS; Gibco, USA), 100 U/mL penicillin, 100 μg/mL streptomycin (Gibco, USA), and 2 mM L-glutamine at 37 °C in a 5% CO_2_ humidified incubator.

### Method details

#### PBMC isolation

PBMCs were isolated from fresh peripheral blood using a human whole-blood mononuclear cell separation medium (TBD Science, Tianjin, China; Cat# LDS1075). After washing with phosphate-buffered saline (PBS; Biosharp), PBMCs were resuspended in RPMI-1640 or DMEM medium (Gibco) supplemented with 10% FBS (Gibco) and antibiotics. Cell viability was assessed using trypan blue staining before downstream experiments.

#### Flow cytometry

Flow cytometry was performed using a FACSCanto II Plus flow cytometer (BD Biosciences, USA). Fluorochrome-conjugated antibodies against CD3, CD45, CD56, TLR2, CD226, PD-1, IFN-γ, and TNF-α were used according to the manufacturers’ instructions. Detailed antibody information is provided in the Key Resources Table. For intracellular cytokine staining, cells were fixed and permeabilized prior to staining.

The flow cytometry validation cohort included 40 participants (*n* = 20 per group). Compensation was performed on single-stained controls. Unstained and isotype controls were included where appropriate. Cell viability was assessed by 7-AAD staining. Data were analyzed using FlowJo software (version 10.8.2, BD Biosciences). The gating strategy consisted of sequential selection of lymphocytes based on forward and side scatter, singlets, 7-AAD-viable cells and CD3^−^CD56^+^NK cells. Expression of TLR2, CD226, PD-1, IFN-γ, and TNF-α was quantified as percentages of positive cells or mean/median fluorescence intensity. Representative gating strategies are shown in Figure S1, following established flow cytometry reporting practices.^34^^,^^35^

#### *In vitro* PBMC stimulation

PBMCs were cultured at a density of 1 × 10^6^ cells/mL in complete DMEM. Cells were stimulated with hippuric acid (B25661, Source Leaf, China; final concentrations of 5 or 10 μM, as indicated), PA (KC004, Kunchuang, China; final concentration of 100 μM), oleic acid (final concentration of 100 μM), or D-glucose (BS099-500g, Biosharp, China; final concentration of 25 mM) to mimic metabolic stress. For the hippuric acid dose-response experiments, PBMCs were treated with 0, 5, or 10 μM hippuric acid, with the corresponding untreated or vehicle-treated cells serving as the 0 μM control. Unless otherwise specified, 10 μM hippuric acid was used in subsequent functional experiments. Additionally, cells were stimulated with PAO (100 μM PA plus 25 mM D-glucose) to establish combined lipid and glucose overload.

For fatty acid stimulation, PA and oleic acid were prepared as fatty acid–BSA conjugates. A matched fatty acid-free BSA vehicle control was included in parallel. For high-glucose stimulation, mannitol was used as the osmotic-matched control. Cells were incubated for 24 h at 37 °C in a 5% CO_2_ humidified incubator. For intracellular cytokine staining, monensin sodium (MultiSciences Biotech, China; Cat# CS0004) was added during the final 4 h of stimulation. PBMCs cultured under the corresponding matched control conditions were used as controls.

For TLR2 agonist stimulation experiment, PBMCs from CHB patients (*n* = 6) were stimulated with Pam3CSK4 (InvivoGen), a synthetic TLR2/TLR1 ligand, at 10 ng/mL for 24 h. Each condition was performed in duplicate wells, and the mean value was used for analysis. NK-cell activation markers and intracellular cytokine production were assessed by flow cytometry.

#### NK cell sorting and cell-intrinsic assay

For NK cell-intrinsic assay, NK cells were purified from PBMCs by FACS using a FACSAria cell sorter (BD Biosciences, USA). NK cells were identified as CD3ˆ-CD56ˆ+ lymphocytes. Sorted NK cells were cultured in complete DMEM and preincubated with a TLR2-neutralizing antibody (InvivoGen, USA; 10 μg/mL) or matched isotype control for 30 min, followed by stimulation with hippuric acid (10 μM) for 24 h. Where indicated, unstimulated NK cells were included as controls. The experiments were performed using cells from 5 independent samples, with 2 technical replicates per condition.

#### NK–LX-2 co-culture assay

To assess the effect of NK-cell activation on HSC readouts, NK cells were purified and stimulated with hippuric acid (10 μM) in the presence or absence of a TLR2-neutralizing antibody (InvivoGen, USA; 10 μg/mL) for 24 h. Activated NK cells were then co-cultured with LX-2 cells at a 1:1 ratio in complete medium for 24 h. Subsequently, LX-2 cells were harvested and stained with mouse monoclonal anti-α-SMA antibody (ab7817, Abcam, UK). The median fluorescence intensity (MFI) of α-SMA was measured by flow cytometry. In addition, co-culture supernatants were collected for measurement of PIIINP and HA using routine clinical assays in the hospital laboratory. Additional control experiments were performed to address baseline NK-LX-2 interactions and potential carryover effects. Untreated CHB-derived NK cells co-cultured with LX-2 cells were used as the baseline co-culture control. To evaluate whether hippuric acid directly affects LX-2 cells, LX-2 cells were exposed to hippuric acid alone under the same culture conditions. To distinguish contact-dependent effects from soluble mediator-associated effects, NK cells and LX-2 cells were also co-cultured using a transwell system under the same cell ratio and culture duration. Where indicated, NK cells were pretreated with hippuric acid in the presence or absence of TLR2-neutralizing antibody before co-culture with LX-2 cells. α-SMA expression in LX-2 cells was assessed by flow cytometry as described above. For the transwell assay, NK cells were seeded into the upper chamber of 0.4-μm transwell inserts, and LX-2 cells were seeded into the lower chamber.

#### RNA sequencing

Total RNA was extracted from FACS-sorted NK cells using the MJzol Animal RNA Isolation Kit, followed by purification with the RNAClean XP Kit and RNase-Free DNase Set (Berry Genomics, China). Exploratory RNA-seq analysis was performed on 7 independent donor samples (CHB, *n* = 4; CHB-MASLD, *n* = 3). Potential batch effects were considered during quality-control inspection. RNA quality and quantity were assessed using the Agilent 2100 Bioanalyzer or Agilent 4200 TapeStation, Qubit 2.0 Fluorometer, and NanoDrop ND-2000 spectrophotometer. Poly(A)+ mRNA was enriched using VAHTS mRNA Capture Beads. Sequencing libraries were prepared with the VAHTS Universal V6 RNA-seq Library Prep Kit for Illumina. Libraries were sequenced on an Illumina NovaSeq 6000 platform to generate 150-bp paired-end reads. After adaptor trimming, quality filtering, removal of reads shorter than 25 bp, and rRNA depletion, clean reads were aligned to the human reference genome (GRCh38) using HISAT2 (v2.0.4). Gene-level raw count matrices were then generated and used for differential expression analysis in DESeq2.^36^ Genes with an adjusted *p* value (false discovery rate, FDR) ≤ 0.05 and an absolute log2 fold change ≥1 were considered as DEGs. Given the limited sample size, the RNA-seq data were interpreted as an exploratory transcriptomic dataset, and the transcriptomic findings were interpreted as hypothesis-generating and used primarily to identify candidate pathways associated with NK-cell activation in CHB-MASLD.

#### Untargeted plasma metabolomics

Plasma samples were collected at enrollment, separated by centrifugation, aliquoted, and stored at −80 °C until analysis. Untargeted metabolomic profiling was performed by a commercial service provider (Biotree, Shanghai, China). A total of 60 plasma samples were included in the metabolomics analysis (CHB, *n* = 31; CHB-MASLD, *n* = 29). Metabolites were extracted from plasma by adding methanol/acetonitrile containing internal standards, and pooled quality control (QC) samples were included throughout the analytical run. Untargeted metabolomic profiling was performed by ultra-high performance liquid chromatography-tandem mass spectrometry (UHPLC-MS/MS) on a Vanquish UHPLC system coupled to a Waters ACQUITY UPLC BEH Amide column and an Orbitrap Exploris 120 mass spectrometer. Data were acquired in both positive and negative electrospray ionization modes. The mobile phase consisted of ammonium acetate/ammonia hydroxide in water and acetonitrile. Raw data were processed for peak detection, alignment, normalization, and metabolite annotation using an XCMS-based in-house R pipeline and BiotreeDB. Multivariate analyses, including principal component analysis (PCA) and OPLS-DA, were performed. Differential metabolites were defined by variable importance in projection (VIP) > 1 and *p* < 0.05. Because this was an untargeted metabolomics analysis, OPLS-DA and VIP-based filtering were used as exploratory approaches to prioritize candidate metabolites associated with CHB-MASLD rather than providing targeted quantitative validation. This analysis was intended for exploratory identification of candidate metabolites associated with CHB-MASLD, and no causal inference was made based on metabolomic association analyses alone.

To validate the differential abundance of metabolites observed in untargeted metabolomic profiling, plasma hippuric acid was further quantified using a targeted UPLC-MS/MS assay performed by the Human Metabolomics Institute (HMI, Shenzhen, China). Samples were processed for metabolite extraction, derivatization, and analysis on an ACQUITY UPLC-Xevo TQ-S system (Waters, Milford, MA, USA), according to a standardized protocol. Quantification was performed using authentic reference standards, external calibration curves, and isotope-labeled internal standards. Raw data were processed using the iMAP platform (version 1.0, HMI). The concentration of hippuric acid was calculated based on calibration curves after internal standard normalization. This targeted assay was used exclusively to validate and quantify the plasma hippuric acid identified by untargeted metabolomics.

#### Quantitative hippuric acid analysis

Plasma hippuric acid levels were quantitatively measured in available plasma samples to provide additional concentration context for the untargeted metabolomic findings and the *in vitro* stimulation experiments. Plasma samples were analyzed using a targeted quantitative assay with calibration standards and quality-control samples. The concentration of hippuric acid was calculated based on the corresponding standard curve and reported as an absolute level. Quantitative data of hippuric acid were used as a validation of metabolomic findings and interpreted together with the untargeted metabolomics results.

#### Clinical outcome definition

Clinical outcomes were analyzed in participants with complete follow-up and event adjudication data. Follow-up started on date of baseline clinical evaluation and ended at the first occurrence of a liver-related event, the last available clinical visit, or censoring. Participants without liver-related events were censored at last follow-up. The primary outcome was progression-free survival (PFS). Liver-related events were defined as progression to advanced fibrosis. Advanced fibrosis was defined as an LSM ≥9.4 kPa assessed by transient elastography, consistent with the established noninvasive thresholds for advanced liver fibrosis in patients with CHB.^37^ Detailed outcome definitions are summarized in Table S4.

### Quantification and statistical analysis

All statistical analyses were performed using GraphPad Prism 10 and R software. Continuous variables were presented as mean ± SD or median (interquartile range), as appropriate. Data distribution was assessed for normality before statistical testing. Comparisons between two independent groups were performed using the unpaired Student’s *t* test (normally distributed data) or the Mann-Whitney U test (non-normally distributed data). Paired comparisons between two conditions or matched cell subsets from the same donor were performed using the paired Student’s *t* test or the Wilcoxon matched-pairs signed-rank test, as appropriate. Comparisons among three or more independent groups were performed using one-way analysis of variance (ANOVA) with appropriate post hoc testing or the Kruskal–Wallis test, as appropriate. Comparisons among three or more matched conditions from the same donors were performed using one-way repeated-measures ANOVA with appropriate multiple-comparison testing or the Friedman test, as appropriate. Correlations were evaluated using Pearson or Spearman correlation analysis depending on data distribution.

To assess whether the associations between NK-cell phenotypes and clinical/metabolic variables are independent of potential confounders, multivariable linear regression analyses were performed after adjustment for age, sex, BMI, antiviral therapy, HBeAg status, and HBV DNA. Kaplan-Meier survival curves were generated and compared using the log rank test. Given the limited sample size and exploratory nature, survival results were interpreted cautiously. All tests were two-sided, and a *p* value <0.05 was considered statistically significant. Where asterisks are used in the figures, significance thresholds are defined as ∗*p* < 0.05, ∗∗*p* < 0.01, ∗∗∗*p* < 0.001, and ∗∗∗∗*p* < 0.0001; ns denotes not significant.

Unless otherwise specified, n represents the number of independent biological samples obtained from individual participants or donors. Technical replicate wells were averaged to generate a single value for each biological sample and were not treated as independent observations in the statistical analyses.
