# Supplementary material for: Hippuric acid contributes to NK-cell activation with dependence on TLR2 in chronic hepatitis B complicated by MASLD
Source: iScience. 2026 Sep 1;29(9):117362. doi: 10.1016/j.isci.2026.117362 (PMC13571118; doi:10.1016/j.isci.2026.117362)
Supplement: Supplementary file 1 [file mmc1.pdf]

iScience, Volume *29*

Supplemental information

Hippuric acid contributes to NK-cell activation with dependence on TLR2 in chronic hepatitis B complicated by MASLD

Luyuan Zhang, Anqi Li, Ke Xu, Siyang Tao, and Meijuan Zheng

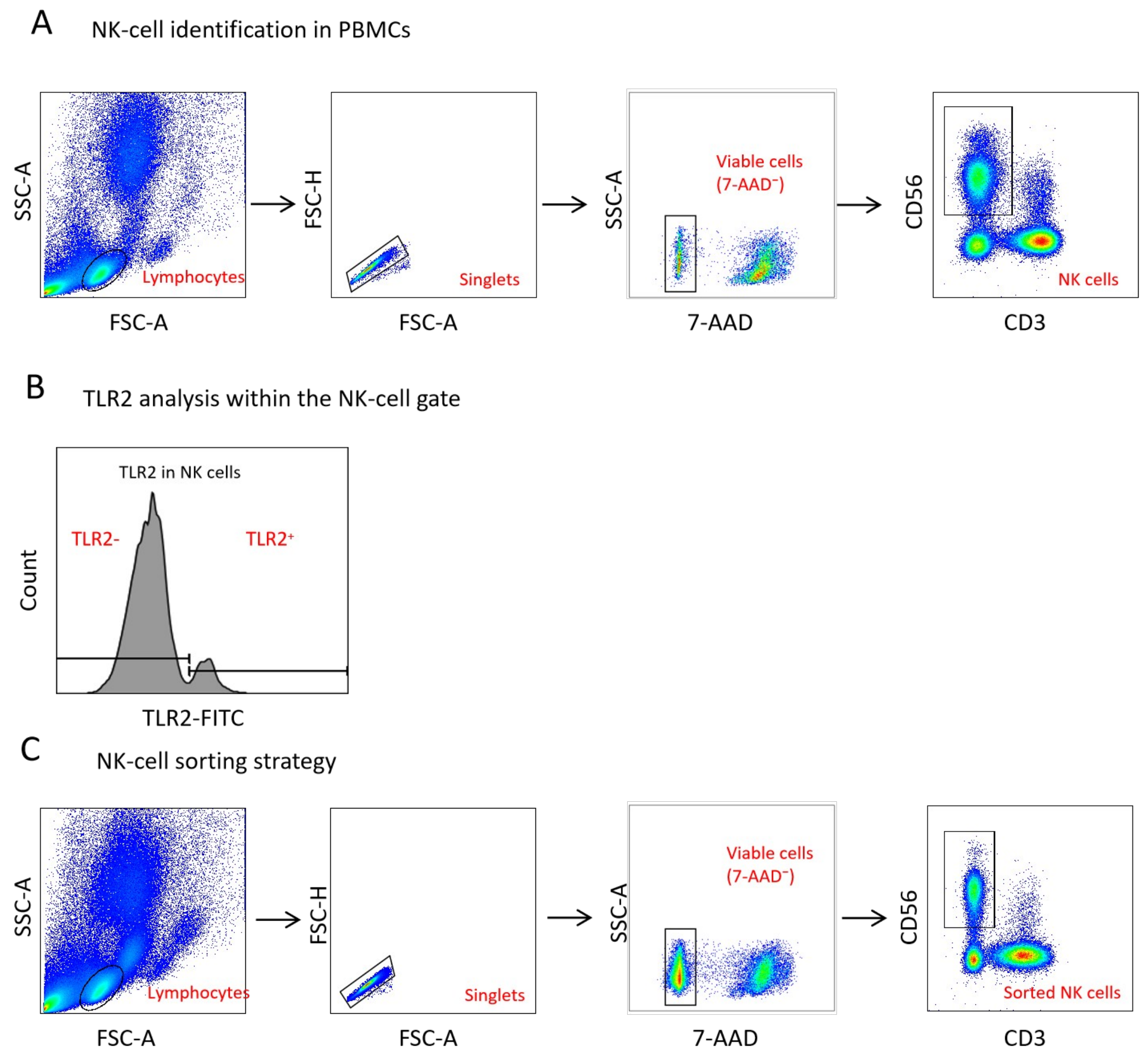

Figure S1. Representative gating strategies for flow cytometric analysis and sorting of NK cells.

(A) Representative gating strategy for identification of $CD3^{-}CD56^{+}$ NK cells in PBMCs.

(B) Representative histogram showing TLR2 expression within the NK-cell gate; no pooled data or error bars are shown.

(C) Representative gating strategy for sorting of $CD3^{-}CD56^{+}$ NK cells.

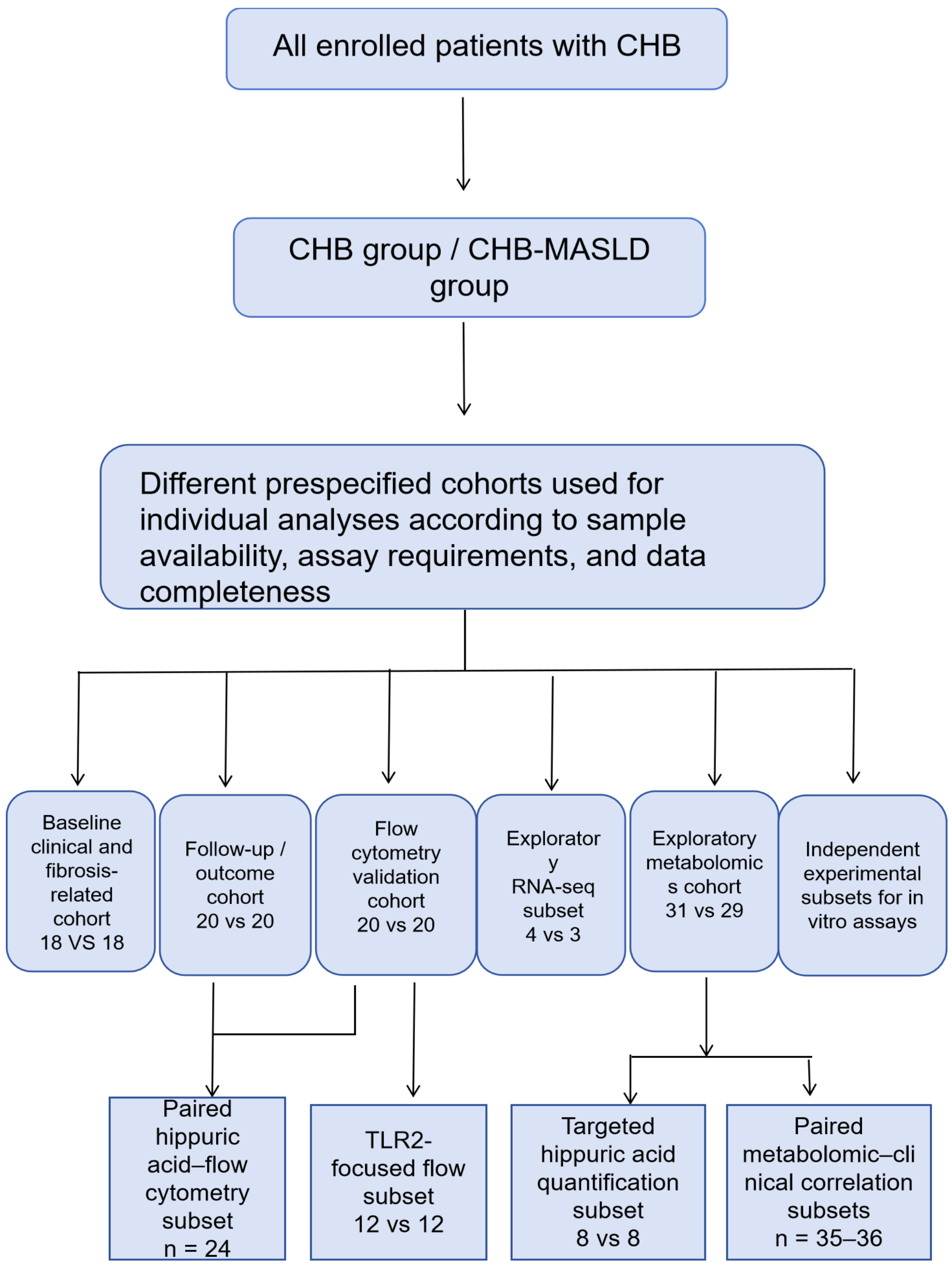

Figure S2. Overview of study design, cohort allocation, and independent experimental subsets.

A schematic overview of cohort allocation is shown. Because complete clinical variables, PBMC samples, RNA preparations, and plasma samples were not available for every participant, different prespecified cohorts were used for individual analyses according to sample availability, assay requirements, and data completeness. Baseline clinical comparisons, fibrosis-related analyses, follow-up outcome analyses, flow cytometric validation, TLR2-focused analyses, exploratory RNA-seq, exploratory metabolomics, and in vitro functional experiments were performed in separate cohorts or independent experimental subsets. Targeted hippuric acid quantification and paired hippuric acid–flow cytometry analyses were performed in additional subsets with available paired measurements. Correlation analyses were restricted to samples with complete paired data for the variables of interest.

A

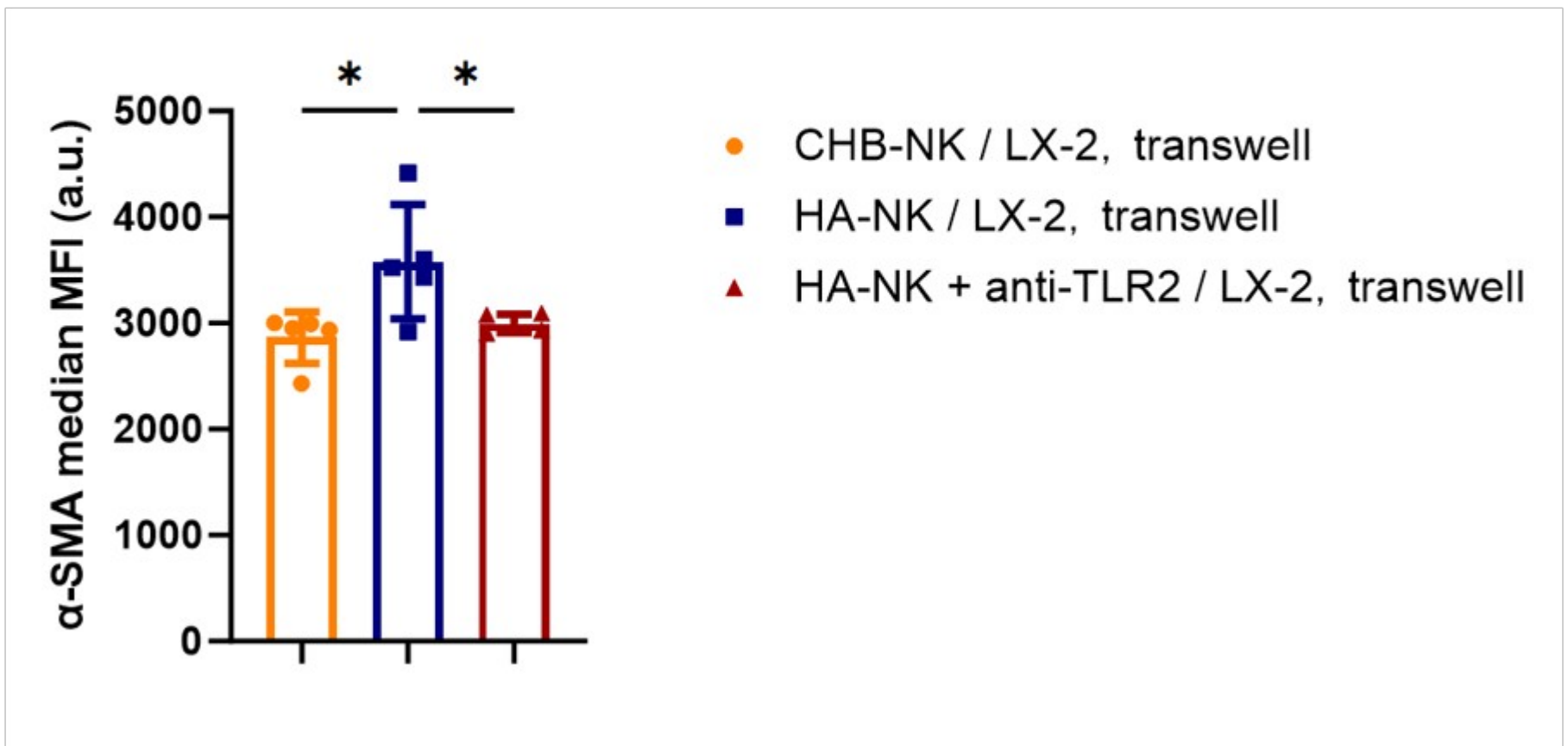

Figure S3. Transwell control experiments in the NK–LX-2 co-culture system.

LX-2 cells were cultured in a transwell system with untreated CHB-derived NK cells, hippuric acid-treated NK cells, or hippuric acid-treated NK cells with TLR2 blockade. α-SMA expression in LX-2 cells was quantified by flow cytometry. Hippuric acid-treated NK cells were associated with increased α-SMA expression, and this effect was attenuated by TLR2 blockade. Experiments were performed using NK cells from 5 independent donors (n = 5). Each data point represents one independent donor. Data are presented as mean ± SD. Comparisons among the three matched conditions were performed using one-way repeated-measures ANOVA followed by Šídák's multiple-comparisons test. *$P < 0.05$.

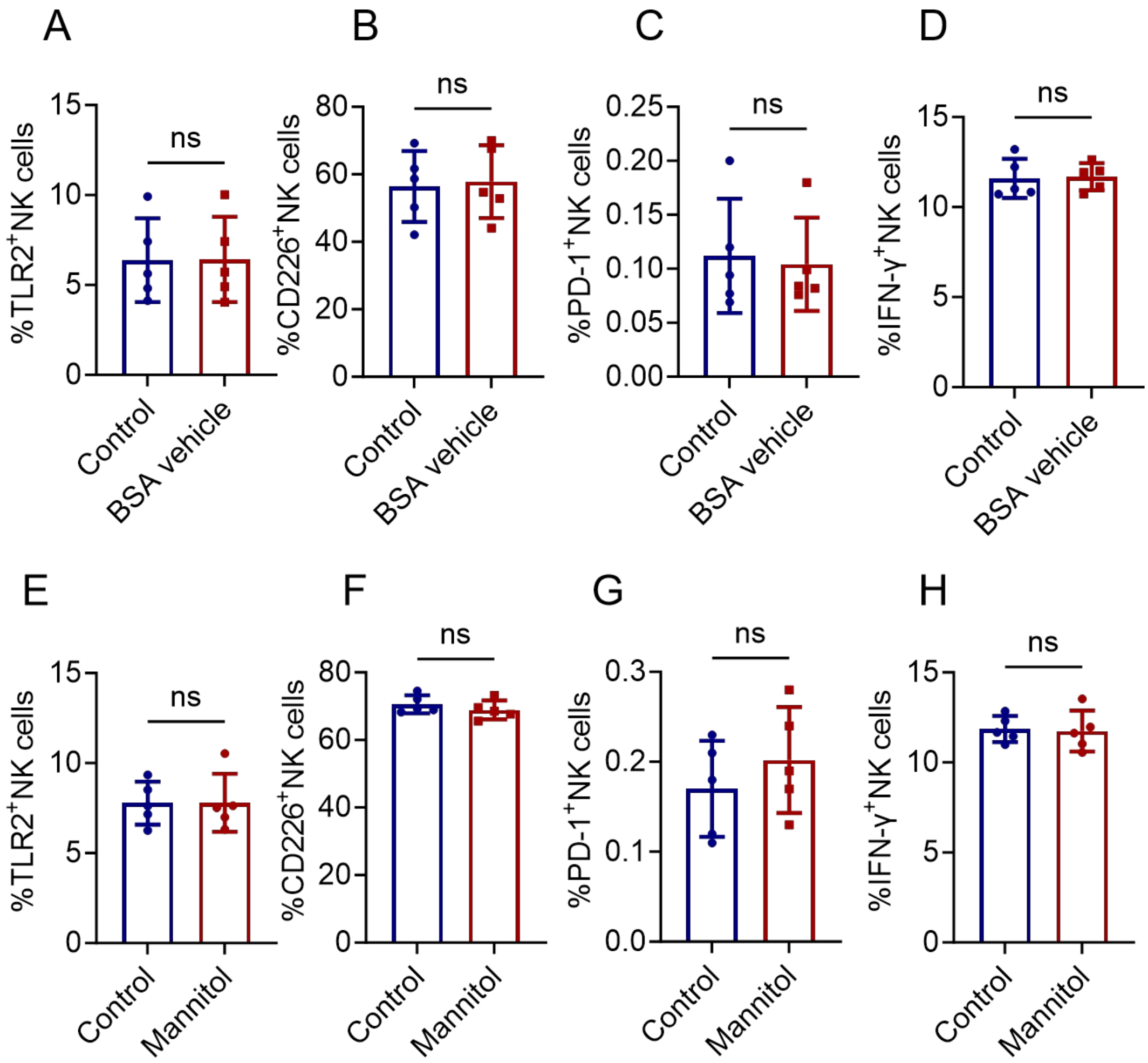

Figure S4. Matched vehicle and osmotic controls do not induce NK-cell activation in vitro. PBMCs were cultured with the matched fatty acid-free BSA vehicle control or mannitol osmotic control under the same conditions used for metabolic stimulation, followed by flow-cytometric analysis of NK-cell phenotype and function. Experiments were performed using PBMCs from five independent donors.

(A–D) Comparison between untreated control and BSA vehicle control, showing the frequencies of (A) TLR2⁺ NK cells, (B) CD226⁺ NK cells, (C) PD-1⁺ NK cells, and (D) IFN-γ⁺ NK cells.(E–H) Comparison between untreated control and mannitol osmotic control, showing the frequencies of (E) TLR2⁺ NK cells, (F) CD226⁺ NK cells, (G) PD-1⁺ NK cells, and (H) IFN-γ⁺ NK cells.Data are presented as mean ± SD. Statistical comparisons were performed using paired Student's t test or Wilcoxon matched-pairs signed-rank test, as appropriate. ns, not significant.

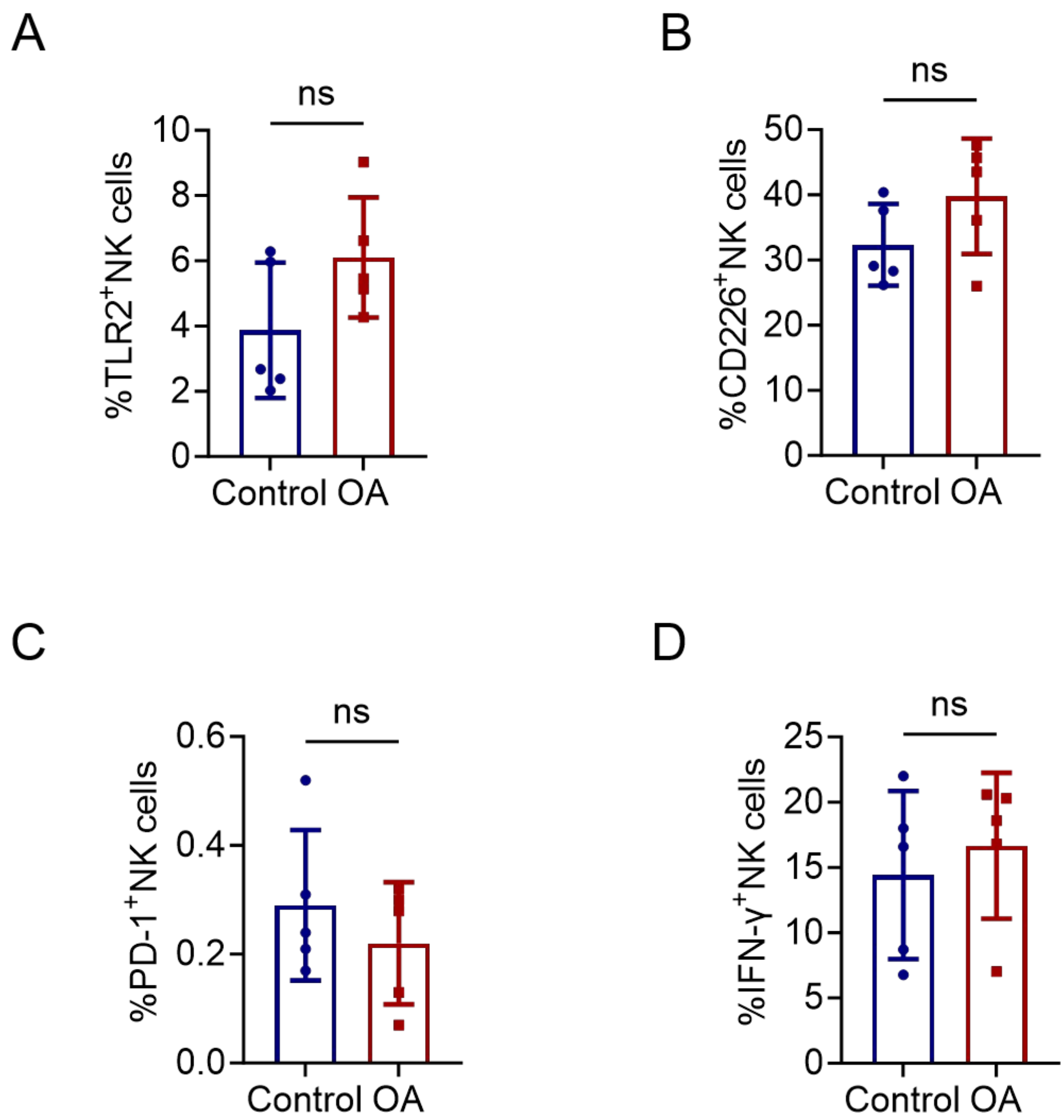

Figure S5. OA induces modest but non-significant changes in NK-cell activation markers compared with the matched vehicle control.

PBMCs were cultured with OA or the matched vehicle control, followed by flow-cytometric analysis of NK-cell phenotype and function. Experiments were performed using PBMCs from 5 independent donors (n = 5), with measurements paired within donors. (A) Frequency of $TLR2^+$ NK cells. (B) Frequency of $CD226^+$ NK cells. (C) Frequency of PD-1^+ NK cells. (D) Frequency of IFN-γ^+ NK cells. Data are presented as mean ± SD. Statistical comparisons were performed using the paired Student's t test or Wilcoxon matched-pairs signed-rank test, as appropriate. ns, not significant. OA, oleic acid.

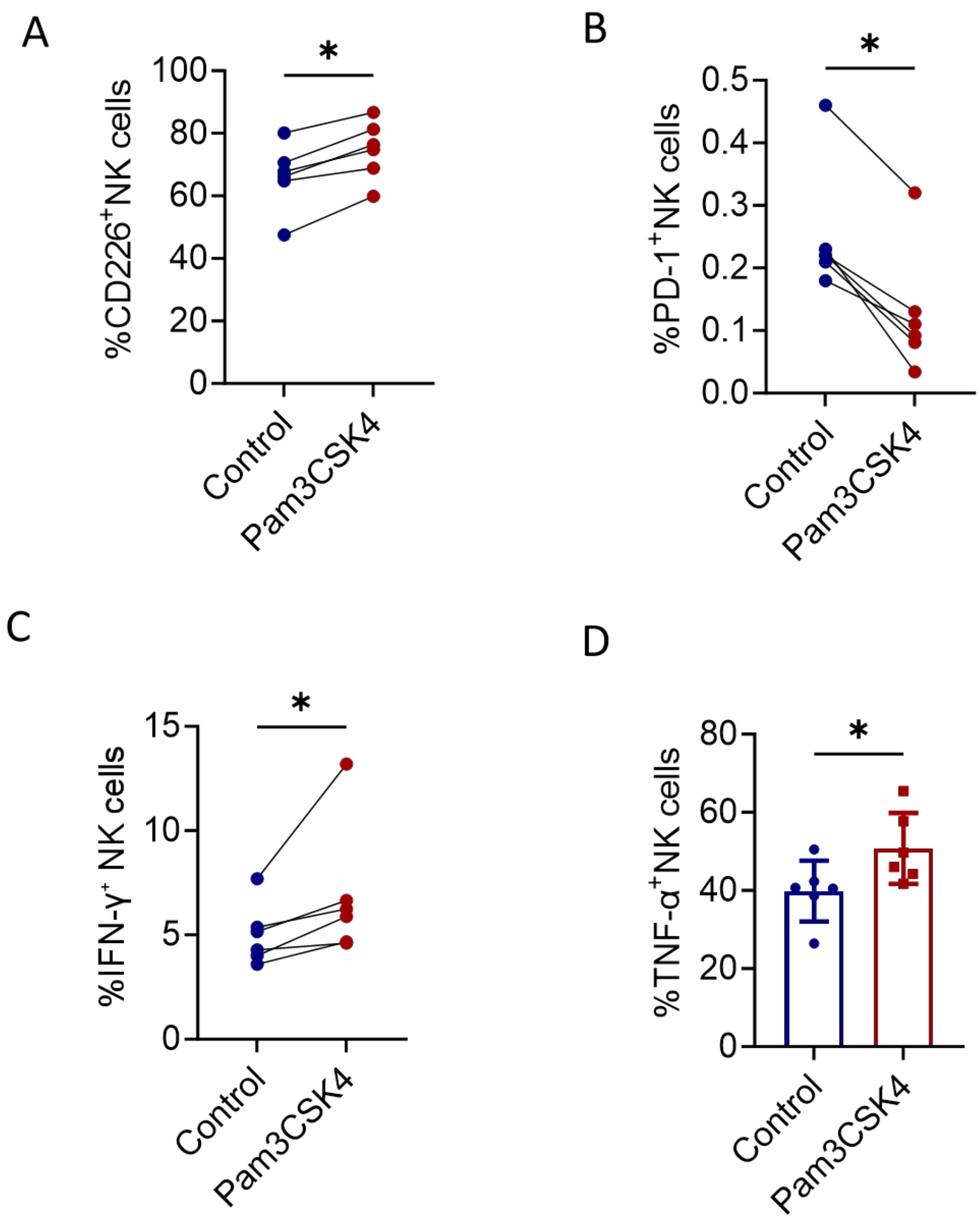

Figure S6. Pam3CSK4 stimulation is associated with enhanced selected NK-cell activation readouts in vitro.

PBMCs from 6 independent donors with CHB ($n = 6$) were stimulated with vehicle control or Pam3CSK4, a synthetic triacylated lipopeptide and TLR2/TLR1 ligand. Duplicate wells were averaged to generate one value per donor, and measurements were paired within donors. NK-cell activation and functional markers were assessed by flow cytometry. Quantification shows the frequencies of (A) $CD226^+$ NK cells, (B) $PD\text{-}1^+$ NK cells, (C) IFN-γ^+ NK cells, and (D) TNF-α^+ NK cells. Pam3CSK4 stimulation increased CD226 expression and cytokine production and reduced PD-1 expression in NK cells. Data are presented as mean ± SD. Statistical comparisons were performed using the paired Student's t test or Wilcoxon matched-pairs signed-rank test, as appropriate. $^*P < 0.05$.

Table S1. Baseline demographic and clinical characteristics of patients with CHB and CHB-MASLD

Variable	CHB (n = 18)	CHB-MASLD (n = 18)	P value
Age, years	45.7 ± 10.3	52.1 ± 9.6	0.089
Male sex, n (%)	10 (55.6)	11 (61.1)	1.000
BMI, kg/m²	22.8 ± 2.1	27.1 ± 2.8	<0.001
ALT, U/L	23.0 (20.0–28.5)	34.0 (26.0–47.0)	0.046
AST, U/L	29.0 (23.0–42.0)	32.0 (23.0–35.5)	0.659
TBil, μmol/L	13.5 (11.5–16.0)	18.0 (13.7–21.7)	0.010
Albumin, g/L	42.8 ± 3.4	41.9 ± 3.8	0.500
Platelet count, ×10^9/L	187.2 ± 37.5	179.3 ± 42.1	0.592
HBV DNA, log10 IU/mL	3.8 (2.5–4.9)	3.5 (2.3–4.6)	0.425
HBeAg positive, n (%)	6 (33.3)	5 (27.8)	1.000
Antiviral therapy, n (%)	11 (61.1)	12 (66.7)	1.000
Fasting glucose, mmol/L	5.1 ± 0.6	6.0 ± 0.7	0.001
HbA1c, %	5.03 (4.87–5.36)	5.66 (5.38–5.88)	0.006
TG, mmol/L	1.42 (0.86–1.50)	2.12 (1.92–2.88)	<0.001
HDL-C, mmol/L	1.20 (1.03–1.50)	0.92 (0.88–0.96)	<0.001
Diabetes mellitus, n (%)	0 (0.0)	4 (22.2)	0.100
Hypertension, n (%)	2 (11.1)	5 (27.8)	0.390
Dyslipidemia, n (%)	3 (16.7)	11 (61.1)	0.009
FIB-4	1.14 (0.99–1.36)	2.25 (2.00–2.60)	<0.001
LSM, kPa	7.2 (6.2–8.5)	10.2 (7.8–15.1)	0.014

Data are presented as mean ± SD, median (IQR), or n (%), as appropriate. Continuous variables were compared using Student's t test or Mann–Whitney U test, and categorical variables were compared using the chi-square test or Fisher's exact test. CHB, chronic hepatitis B; MASLD, metabolic dysfunction-associated steatotic liver disease; BMI, body mass index; ALT, alanine aminotransferase; AST, aspartate aminotransferase; TBil, total bilirubin; HBV, hepatitis B virus; HBeAg, hepatitis B e antigen; HbA1c, glycated hemoglobin; TG, triglycerides; HDL-C, high-density lipoprotein cholesterol; FIB-4, fibrosis-4 index; LSM, liver stiffness measurement.

Table S2. Cohort allocation and sample size used for each analysis

Figure / panel	Analysis content	CHB n	CHB-MASLD n	Source cohort / subset	Inclusion criteria	Notes
Figure 1A–B, F–J	Clinical and metabolic comparisons	18	18	Expanded clinical cohort	Complete clinical data	Used for updated Figure 1 analyses
Figure 1C	Fibrosis stage distribution	20	20	Fibrosis-related subset	Available fibrosis staging data	
Figure 1D–E	Clinical outcome analyses	20	20	Follow-up cohort	Complete follow-up and event adjudication data	See event definition in Table S4 / Methods
Figure 2A–C, F–I	Peripheral NK-cell phenotyping and activation analyses	20	20	Flow cytometry validation cohort	Available PBMC samples and complete staining panels	Independent validation cohort
Figure 2D–E	Exploratory RNA-seq analyses	4	3	Exploratory RNA-seq subset	Sorted NK-cell samples meeting RNA quality-control criteria	Exploratory transcriptomic dataset
Figure 3A	Exploratory RNA-seq heatmap	4	3	Exploratory RNA-seq subset	Same as above	Exploratory transcriptomic dataset
Figure 3B	Frequency of $TLR2^{+}$NK cells	12	12	TLR2-focused flow subset	Complete TLR2 panel measurements	
Figure 3C	TLR2 expression in NK/T-like cells, T cells, and B cells	5	5	Lymphocyte-subset flow cytometry subset	Complete staining data for all indicated lymphocyte subsets	
Figure 3D–G	$TLR2^{-}$ versus $TLR2^{+}$NK cells	—	12	TLR2-focused flow cytometry subset	Complete marker data	CHB-MASLD-only analysis

Figure / panel	Analysis content	CHB n	CHB-MASLD n	Source cohort / subset	Inclusion criteria	Notes
Figure 3H–K	Clinical correlations	—	—	Paired flow–clinical subset	Complete paired data	n = 24 total
Figure 4A–F	Metabolic-stress experiments	—	—	Independent PBMC experimental subset	Sample availability and assay requirements	n = 10 donors
Figure 5A–D	Exploratory untargeted metabolomics	31	29	Exploratory metabolomics cohort	Plasma samples passing quality-control criteria	Exploratory metabolomic dataset
Figure 5E	Quantitative hippuric acid	8	8	Targeted hippuric acid quantification subset	Available plasma samples	
Figure 5F–H	Clinical correlations	—	—	Paired metabolomic–clinical subset	Complete paired data	n = 35, 36, and 35
Figure 6A	Hippuric acid–NK-cell correlation	—	—	Paired plasma–flow subset	Complete paired data	n = 24 total
Figure 6B–C	Dose-response experiments	—	—	PBMC experimental subset	Complete paired data	n = 5 donors
Figure 6D	Hippuric acid stimulation	—	—	PBMC experimental subset	Complete data	n = 15 donors
Figure 6E	$TLR2^{-}$ versus $TLR2^{+}$NK cells	—	—	Matched NK-cell subset	Complete paired data	n = 10 donors
Figure 6F	PBMC TLR2 blockade	—	—	PBMC blockade subset	Complete data	n = 6 donors
Figure 6G	Sorted NK-cell TLR2 blockade	—	—	Sorted NK-cell subset	Complete data	n = 5 donors
Figure 6H–K	NK–LX-2 co-culture	—	—	Co-culture subset	Complete data	n = 5 donors
Figure S3	Transwell experiment	—	—	Transwell subset	Complete data	n = 5 donors

Figure / panel	Analysis content	CHB n	CHB-MASLD n	Source cohort / subset	Inclusion criteria	Notes
Figure S4	Vehicle and osmotic controls	—	—	PBMC control subset	Complete paired data	n = 5 donors
Figure S5	Oleic acid experiment	—	—	PBMC experimental subset	Complete paired data	n = 5 donors
Figure S6	Pam3CSK4 experiment	6	—	CHB PBMC subset	Complete paired data	n = 6 donors

Note: Different cohorts or experimental subsets were used according to sample availability and data completeness. Correlation analyses included participants with complete paired measurements. Unless otherwise specified, n represents independent participants or donors. Technical replicates were averaged before statistical analysis.

Table S3. Multivariable linear regression analyses of $TLR2^{+}$NK-cell frequency in CHB and CHB-MASLD

Dependent variable	Predictor	Adjusted covariates	Regression coefficient (B)	95% CI	P value
$TLR2^{+}$ NK-cell frequency	CHB-MASLD vs. CHB	Age, sex, BMI, antiviral therapy, HBV DNA, HBeAg status	5.2	1.4–9.0	0.010
$TLR2^{+}$ NK-cell frequency	Triglycerides (TG)	Age, sex, BMI, antiviral therapy, HBV DNA, HBeAg status	2.7	0.53–4.87	0.018
$TLR2^{+}$ NK-cell frequency	Fasting glucose (GLU)	Age, sex, BMI, antiviral therapy, HBV DNA, HBeAg status	1.5	0.10–2.90	0.037

Dependent variable	Predictor	Adjusted covariates	Regression coefficient (B)	95% CI	P value
TLR2+ NK-cell frequency	Liver stiffness measurement (LSM)	Age, sex, BMI, antiviral therapy, HBV DNA, HBeAg status	0.6	0.09–1.11	0.024

Note: Each predictor was evaluated in a separate multivariable linear regression model adjusted for age, sex, BMI, antiviral therapy, HBV DNA, and HBeAg status. Analyses included 24 participants with complete paired clinical and flow cytometry data and were considered exploratory. regression coefficients (B), 95% confidence intervals, and P values were obtained from the corresponding regression models.

Table S4. Definition of liver-related events and follow-up information used for clinical outcome analyses

Analysis item	Definition / description
Outcome analysis cohort	Patients with complete follow-up data who were included in the Kaplan–Meier and cumulative incidence analyses
Follow-up start point	Baseline clinical evaluation
Follow-up end point	Last clinical follow-up, occurrence of a liver-related event, or censoring at the last available visit
Censoring rule	Participants without liver-related events were censored at the date of last follow-up
Primary outcome	Fibrosis progression-free survival
Liver-related events	First progression from a baseline LSM < 9.4 kPa to an LSM ≥ 9.4 kPa during follow-up, as assessed by transient elastography.
Baseline advanced fibrosis handling	Participants with advanced fibrosis at baseline, defined as LSM ≥ 9.4 kPa, were excluded from the fibrosis progression-free survival analysis.
Event adjudication	Events were identified from clinical follow-up records and adjudicated according to predefined criteria
Number of events in CHB group	7
Number of events in CHB-MASLD group	11

Analysis item	Definition / description
Median follow-up duration	9 months
Statistical methods	Kaplan–Meier analysis with the log-rank test for fibrosis progression-free survival; the cumulative incidence of fibrosis progression was estimated as 1 minus the Kaplan–Meier event-free survival estimate.

Note： Only participants with complete follow-up and event adjudication data were included in the outcome analyses.
